# Mucin1 and Mucin16: Therapeutic Targets for Cancer Therapy

**DOI:** 10.3390/ph14101053

**Published:** 2021-10-17

**Authors:** Dong-Hee Lee, Seunghyun Choi, Yoon Park, Hyung-seung Jin

**Affiliations:** 1 Department of Convergence Medicine, Asan Institute for Life Sciences, Asan Medical Center, University of Ulsan College of Medicine, Seoul 05505, Korea; dhlee3342@gmail.com; 2 Center for Theragnosis, Biomedical Research Institute, Korea Institute of Science and Technology (KIST), Seoul 02792, Korea; 221508@kist.re.kr

**Keywords:** mucin, MUC1, MUC16, immunotherapy, cancer vaccine, CAR (chimeric antigen receptor), ADC (antibody-drug conjugate)

## Abstract

The mucin (MUC) family is a group of highly glycosylated macromolecules that are abundantly expressed in mammalian epithelial cells. MUC proteins contribute to the formation of the mucus barrier and thus have protective functions against infection. Interestingly, some MUC proteins are aberrantly expressed in cancer cells and are involved in cancer development and progression, including cell growth, proliferation, the inhibition of apoptosis, chemoresistance, metabolic reprogramming, and immune evasion. With their unique biological and structural features, MUC proteins have been considered promising therapeutic targets and also biomarkers for human cancer. In this review, we discuss the biological roles of the transmembrane mucins MUC1 and MUC16 in the context of hallmarks of cancer and current efforts to develop MUC1- and MUC16-targeted therapies.

## 1. Introduction

Mucins are large and highly glycosylated proteins that provide hydration and lubrication to the epithelial luminal surface of body duct, including the gastrointestinal, respiratory, and reproductive tracts. Mucins also act as a physical barrier against various pathogens in the epithelium [1,2]. Mucins are classified into two types: secreted or transmembrane (membrane-bound) mucins. Secreted mucins are comprised of gel-forming and non-gel-forming mucins, and include MUC2, MUC5AC, MUC5B, MUC6, MUC7, MUC8, MUC9 (OVGP1), and MUC19. Transmembrane mucins, comprising a single membrane-spanning domain and a cytoplasmic domain, have been identified as MUC1, MUC3A, MUC3B, MUC4, MUC12, MUC13, MUC14 (endomucin), MUC15, MUC16, MUC17, MUC20, MUC21 (epiglycanin), and MUC22 [3,4].

MUC1 was the first mucin to be identified [5]. After its initial identification in human pancreatic cancer [6,7], MUC1 expression has been detected in most epithelial cells [8]. In addition, it has been reported that MUC1 is overexpressed in a variety of cancer tissues including in pancreatic, breast, ovarian, lung, and colon carcinomas [9]. The aberrant expression of MUC1 can induce a loss of polarity of epithelial cells and altered downstream signals through its cytoplasmic domain [2,10]. Ectopically expressed MUC1 in rat fibroblasts induces their cellular transformation and tumor formation in the mouse [11]. In addition, a series of findings have indicated that MUC1 is an attractive target for anti-cancer treatment [12,13,14,15].

MUC16 (also known as carbohydrate antigen 125, CA125) is the largest transmembrane mucin and is normally expressed in the epithelium of the upper respiratory tract, ocular surface, mesothelium lining body cavities (pleural, peritoneal, and pelvic cavities), internal organs, and male and female reproductive organs [16,17,18]. Since MUC16 is known to be overexpressed on the surface of ovarian cancer cells and cleaved/shed into blood, it is a well-established serum biomarker for ovarian cancer [19]. Even though signaling pathways via the MUC16 cytoplasmic domain are largely unknown, a strong correlation between the serum CA125/MUC16 level and ovarian cancer prognosis also suggests that MUC16 is a potential therapeutic target for the treatment of ovarian cancer [20].

A variety of therapeutic agents have been explored to target onco-mucins for cancer treatment. The extracellular domain of membrane-bound mucins on the surface of cancer cells can be a potential target for monoclonal antibody-based cancer therapeutics [21,22]. It is also feasible to modulate signaling pathways directly through the cytoplasmic domain of mucins or to boost the host immune reaction against tumor via vaccinations with mucin antigens [22]. The purpose of this review is to summarize both intrinsic and extrinsic roles of MUC1 and MUC16 in modulating tumorigenesis and the recent advances made in exploiting the therapeutic potential of these transmembrane mucins.

## 2. Transmembrane Mucin Structure

### 2.1. Core Structural Characteristics

Transmembrane mucins are type I membrane proteins with a single membrane span. Their N-terminal extracellular region comprises a tandem-repeat (TR) domain, SEA (sea urchin sperm protein enterokinase and agrin) domain, and/or an EGF (epidermal growth factor)-like domain [2]. The TR domain contains a variable number of repeated amino acid sequences and is rich in serine, threonine, and proline (S/T/P). These S/T/P residues are the sites for *O*-linked N-acetylgalactosamine (GalNAc) addition to initiate further *N*-linked glycosylation chain reactions [23]. The TR domain underlies the physical and chemical features of these molecules, such as lubrication or immune protection, due to its highly glycosylated structure. The SEA domain has a highly conserved cleavage site located close to the outside of the cell membrane. Proteolytic cleavage of transmembrane mucins divides them into an N-terminal subunit containing an extracellular TR and C-terminal subunit harboring the transmembrane and cytoplasmic domains. These two subunits can form a non-covalent and stable complex [10]. The EGF-like domain shares sequence homology with growth factors such as EGF or cytokines and interacts with growth factor receptors such as the ErbB receptor [24]. The cytoplasmic domain of transmembrane mucins is relatively short. Due to the presence of known protein-binding motifs and tyrosine residues for phosphorylation, this domain is considered to have a role in signal transduction. The specific structures of MUC1 and MUC16 are described below in more detail.

### 2.2. Structure of MUC1

**Figure 1:**
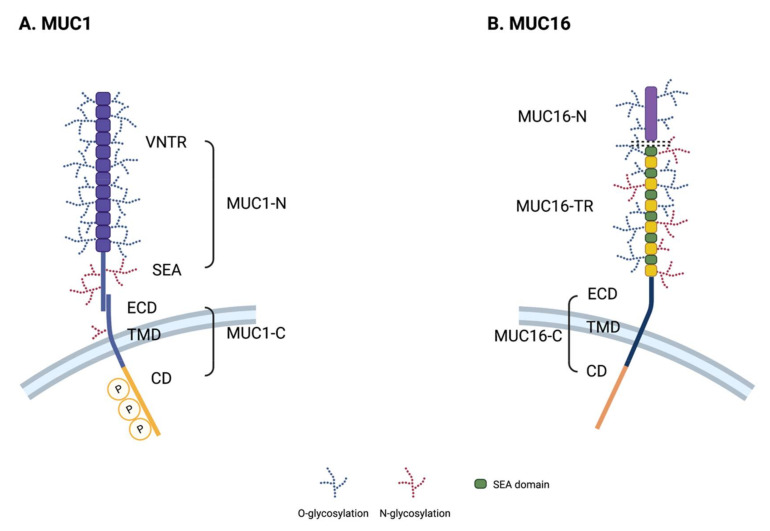
Schematic representation of the MUC1 and MUC16 structures. (**A**) MUC1 is a stable heterodimeric complex with an N-terminal subunit (MUC1-N) and C-terminal subunit (MUC1-C). The variable number tandem repeat (VNTR) region in MUC1-N is composed of a 20 amino acid repeat sequence that is extensively *O*-glycosylated at the serine and threonine residues. SEA domain is auto-cleaved and non-covalently linked to the extracellular domain (ECD) of the MUC1-C subunit. MUC1-C is anchored in the plasma membrane of cells via its transmembrane domain (TMD). The cytoplasmic domain (CD) of MUC1 contains potential binding motifs for various signaling proteins with phosphorylation sites. (**B**) MUC16 is a single transmembrane glycoprotein consisting of a large N-terminal domain (MUC16-N) and tandem repeat domain (MUC16-TR) that is interspersed with an SEA domain and C-terminal domain (MUC16-C).

MUC1, also known as EMA (tumor-associated epithelial membrane antigen) or CD227, is a large, glycosylated protein with expected molecular weights ranging from 120 to 500 kDa, depending on the glycosylation status. A variable number tandem repeat (VNTR) domain in MUC1 consists of 20–125 repeats of a 20 amino acid sequence (PAPGSTAPPAHGVTSAPDTR). MUC1 also contains a 110 amino acid long single SEA, short transmembrane region, and 74 amino acids of cytoplasmic region (Figure 1A). Its cytoplasmic domain has several short (4~9 amino acid long) protein-binding motifs that facilitate its interaction with GSK3β (glycogen synthase kinase 3 beta), β-catenin, GRB2 (growth factor receptor-bound protein 2), SRC, and ESR1 (estrogen receptor 1) [25,26,27,28,29,30]. It also possesses a p53 binding region, which is a relatively long 37 amino acid sequence [31]. MUC1 has several isoforms, some of which do not have TR regions, such as the J13 or Y variants [32,33,34].

### 2.3. Structure of MUC16

MUC16 is the largest transmembrane mucin and comprises ~14,000 amino acids with molecular weights ranging from 1.5 to 5 MDa. MUC16 contains three major domains: an N-terminal domain (MUC16-N), a tandem repeat domain (MUC16-TR), and a C-terminal domain (MUC16-C) (Figure 1B). Its N-terminal domain contains multiple serine-rich regions inside of a ~12,000 amino acid long threonine-rich region, which is exclusively *O*-glycosylated. The TR domain contains 12~60 repeats of 156 amino acids with an interspersed SEA domain, which harbors both *O*-linked and *N*-linked glycosylation sites [35]. Unlike MUC1, MUC16 has been known to contain 16 SEA modules [36]. The C-terminal domain of MUC16 comprises an extracellular domain, short transmembrane region, and a 32 amino acid cytoplasmic domain. The cytoplasmic domain of MUC16 contains a polybasic amino acid motif (RRRKK) that associates with ezrin/radixin/moesin (ERM) actin-binding proteins [37]. The MUC16 cytoplasmic domain also contains several serine/threonine/tyrosine residues; the third tyrosine residue of the cytoplasmic domain is known to be phosphorylated by c-Src kinase [35,38].

## 3. The Role of Transmembrane Mucins in Tumorigenesis

**Figure 2:**
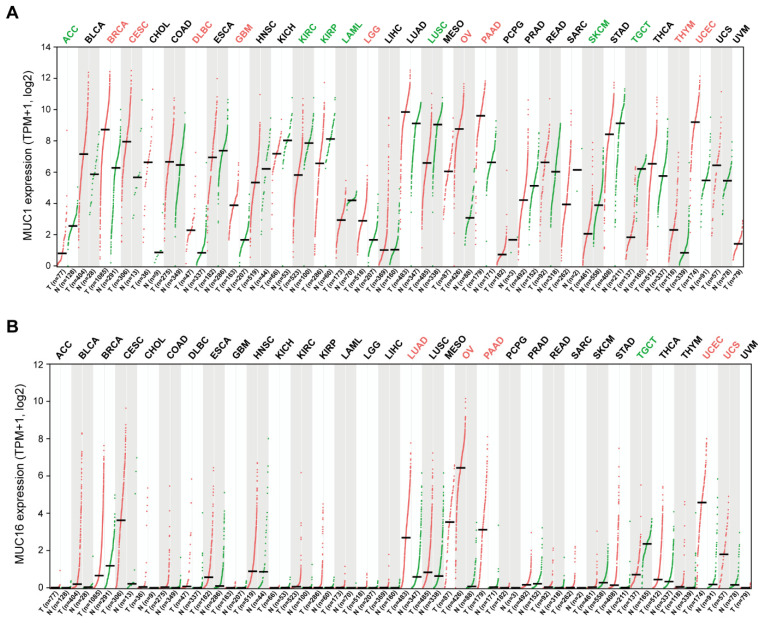
*MUC1* and *MUC16* mRNA expression in tumor and normal tissues. Pan-cancer expression analysis of *MUC1* and *MUC16* genes was conducted using the GEPIA2 web server [55]. Tumor tissues (T, red dots) represent TCGA tumors. Normal tissues (N, green dots) represent TCGA and GTEx normal tissues. Expression values are presented as log-normalized transcripts per million (TPM) with median values (horizontal black bar). The red and green colors of the cancer type abbreviations denote that *MUC1* or *MUC16* gene expression in significantly higher or lower in these tumor tissues compared with normal tissues. (**A**) *MUC1* is overexpressed in tumors of the breast (BRCA), cervix (CESC), brain (GBM, LGG), B-cell (DLBC), pancreas (PAAD), ovary (OV), thymus (THYM), and uterus (UCEC). (**B**) *MUC16* is overexpressed in tumors of the lung (LUAD), ovary (OV), pancreas (PAAD), and uterus (UCEC and UCS).

Transmembrane mucins have long been considered promising anti-cancer targets because they are abnormally overexpressed in various carcinomas of the lung [39,40,41], breast [42,43,44], pancreas [45,46,47], digestive tract [48,49,50,51], and ovary [52,53]. Among the transmembrane mucin family, MUC1 and MUC16 are the most well-studied in terms of their clinical significance in tumorigenesis. To determine differences of *MUC1* and *MUC16* expression in tumor and normal tissues, the *MUC1* and *MUC16* mRNA levels in multiple types of tumor tissues were analyzed in the cancer genomics database TCGA (The Cancer Genome Atlas) (Figure 2). *MUC1* expression was higher in BRCA (breast invasive carcinoma), CESC (cervical squamous cell carcinoma and endocervical adenocarcinoma), GBM (glioblastoma), LGG (brain lower grade glioma), DLBC (lymphoid neoplasm diffuse large B-cell lymphoma), PAAD (pancreatic adenocarcinoma), OV (ovarian serous cystadenocarcinoma), THYM (thymoma), and UCEC (uterine corpus endometrial carcinoma) compared with adjacent normal tissues. On the contrary, lower expression was observed in ACC (adrenocortical carcinoma), KIRC (kidney renal clear cell carcinoma), KIRP (kidney renal papillary cell carcinoma), LAML (acute myeloid leukemia), LUSC (lung squamous cell carcinoma), SKCM (skin cutaneous melanoma), and TGCT (testicular germ cell tumors). *MUC16* expression was significantly higher in the LUAD (lung adenocarcinoma), OV, PAAD, UCEC, and UCS (uterine carcinosarcoma) compared with adjacent normal tissues.

Tumorigenesis is a complex process involving a variety of events inside and outside of transformed cells. Abnormal alterations of these biological events are well-characterized as “hallmarks of cancer” [54]. Although each hallmark of cancer is initiated by a specific gene, some genes are known as multifunctional master regulators of several hallmarks. Many reports have suggested that transmembrane mucins may play multiple roles in tumorigenesis and tumor progression. We here summarize the detailed tumorigenic roles of MUC1 and MUC16 in the context of cancer hallmarks.

### 3.1. Uncontrolled Proliferation

One of the essential hallmarks of cancer cells is an unlimited proliferative potential sustained by abnormal growth signaling pathways. Constitutive activation of growth factor signaling is conferred by oncogenic mutations or by the overexpression of receptor tyrosine kinases (RTKs), followed by protein–protein interactions that transmit downstream signals [56]. The cytoplasmic domain of MUC1 has several protein-binding motifs and phosphorylation sites that are important for protein–protein interactions. RTKs are known to interact directly with MUC1 for oncogenic signaling. ErbB is a family of RTKs consisting of ErbB1 (also known as EGF receptor), ErbB2 (also known as HER2/Neu), ErbB3, and ErbB4. MUC1 interacts with all of the ErbB family receptors to transmit oncogenic signaling reciprocally. The cytoplasmic domain of MUC1 was found in breast cancer cell lines to be phosphorylated by ErbB1 at the YEKV motif, resulting in c-Src and β-catenin recruitment and downstream signaling [57]. Reciprocally, MUC1 also potentiates ErbB signaling. The increased expression of MUC1 activates MAPK signaling through a physical interaction with ErbB1 and inhibition of ErbB1 degradation in breast cancer cells [58,59]. MUC1 also binds to fibroblast growth factor receptor 3 (FGFR3), another key RTK in tumorigenesis. Upon FGF1 ligand stimulation, FGFR3 interacts with MUC1 and phosphorylates the YEKV motif of the MUC1 cytoplasmic domain. This phosphorylated MUC1 forms a complex with β-catenin and translocates into the nucleus [60]. MUC1 also increases cytosolic β-catenin levels by inhibiting GSK3β-mediated phosphorylation and degradation. A serine-rich motif (SRM) of MUC1 interacts directly with the Armadillo repeats of β-catenin [61].

Estrogen receptor alpha (ERα) is a nuclear receptor that acts as an oncogene in a specific type of hormone-dependent (ER+) breast cancer. The nuclear localization and dimerization of ERα by estrogen stimuli activates the transcription of genes that contain an estrogen response element (ERE) within their regulatory regions. MUC1 interacts with the DNA-binding domain of ERα directly and thereby stabilizes ERα by blocking proteasomal degradation, resulting in enhanced ERα response gene transcription and the proliferation of breast cancer cells [29].

### 3.2. Evading Cell Death and Resistance to Stress

Another important hallmark of cancer is resistance to apoptotic cell death. Fast proliferating cancer cells face various stress conditions arising from internal (e.g., DNA replication, protein translation and degradation, and mitochondrial respiration) or external (e.g., tumor microenvironment and anti-cancer drugs) factors. Cellular stress pathways usually accompany apoptotic signals to eliminate damaged or transformed cells. Cancer cells evade stress-induced apoptosis through various mechanisms, and MUC1 has a protective role that contributes to this survival. First, MUC1 attenuates the genotoxic stress induced by DNA damage from DNA replication mechanisms or the actions of anti-cancer drugs. MUC1 regulates p53-dependent gene transcription through its direct association with the p53 regulatory domain and p53-responsive element. Upon treatment of cancer cells with DNA damage inducing agents (e.g., cisplatin and etoposide), MUC1 promotes transcription of growth arrest genes and suppresses p53-dependent apoptotic genes, thereby promoting the survival of these cells upon exposure to anti-cancer agents [31]. On the other hand, MUC1 directly exploits the drug efflux system through the transcription of multidrug resistance (MDR) genes, which has been reported to protect both lung and pancreatic cancer cells from chemotherapeutics [62,63].

MUC1 attenuates mitochondrial apoptotic factors such as cytochrome c or Bcl-xL (B-cell lymphoma-extra-large), protecting cancer cells from anti-cancer genotoxins such as cytarabine, gemcitabine, and cisplatin [64,65]. Upon genotoxic stress, c-Abl combined with 14-3-3 protein localizes in the nucleus where it activates the proapoptotic c-Jun N-terminal kinase (JNK) pathway [66]. MUC1 blocks this nuclear translocation of the c-Abl protein and thereby inhibits the apoptotic response to genotoxic anti-cancer drugs [67]. The constitutive activation of the NF-κB pathway is another anti-apoptotic mechanism activated by genotoxic stress. Oncogenic MUC1 promotes the phosphorylation and degradation of IκBα via an association with IKKβ and IKKγ [68].

MUC1 provides survival advantage to cancer cells by scavenging oxidative stress. MUC1 dephosphorylates and activates FOXO3a, which is tightly regulated by the PI3K/AKT pathway. FOXO3a activation induces its nuclear localization and the subsequent transcription activation of ROS scavenging genes. The stable downregulation of MUC1 has been shown to increase the intracellular ROS levels and sensitize breast cancer cells to ROS-induced necrosis [69].

Cancer cells are exposed to Fas (CD95/APO-1) and the Fas ligand (FasL) mediated apoptosis pathway when engaged by tumor-killing lymphocytes. The MUC1 cytoplasmic domain binds to Fas-associated death domain (FADD) and regulates FADD-induced caspase-8 activation. Hence, MUC1-high expressing cancer cells can evade the extrinsic apoptosis pathway [70].

MUC16 is also known to play an anti-apoptotic role in cancer cells. The ectopic expression of the c-terminal domain of MUC16 induces cisplatin resistance in ovarian cancer cells [71]. Lakshmanan et al. have also previously demonstrated a chemoresistant role of MUC16 in lung cancer cells that is mediated through the suppression of p53 [72]. Although its binding partners and precise molecular mechanisms underlying the resistance phenotypes are still unknown, the cytoplasmic domain of MUC16 is believed to have a signaling role that is comparable to MUC1.

### 3.3. Reprogramming Energy Metabolism

Since aerobic glycolysis was proposed as a unique glucose metabolic process in cancer, reprogramming pathways for acquiring nutrients and their subsequent metabolism are also an accepted cancer hallmark [54,73]. The altered expression of mucins in various cancer tissues is additionally suggested as a mediator of this reprogramming of energy metabolism. Chaika et al. demonstrated in an earlier study that MUC1 increases the glucose metabolism levels in pancreatic cancer. MUC1 overexpression also showed an association with increased glucose uptake, and with HIF-1α, GLUT1, and LDHA protein expression, in an orthotopic mouse model of pancreatic cancer. MUC1, together with HIF-1α, binds to the hypoxia response element (HRE) in the promoter region of the key glycolysis enzymes *ENO1* and *PGM2*. Furthermore, a prior metabolomics study has illustrated a global metabolic shift, including amino acid metabolism and the TCA cycle, as well as glycolysis, in MUC1-overexpressing pancreatic cells [74]. MUC16 also has a similar role in metabolic reprogramming in pancreatic cancer through the mTOR (mammalian target of rapamycin) and c-MYC pathways [75]. Another study has suggested a role of the cytoplasmic domain of MUC1 in these processes. Rat fibroblasts transformed via the ectopic expression of MUC1 show altered glucose uptake and lactate production. MUC1 stimulates pyruvate kinase M2 (PKM2), a key mediator of anaerobic glycolysis, through a direct association [76]. MUC1 also contributes to altering the pentose phosphate pathway (PPP) and the nucleotide metabolism of pancreatic cancer cells. Inducing sufficient DNA damage is necessary to kill cancer cells during radiation therapy. However, cancer cells can be protected from DNA damage stress by an upregulated PPP and stronger nucleotide metabolism to secure a larger nucleotide pool [77]. High MUC1 expression also reduces cancer cell sensitivity to radiation in vitro and in vivo. This resistance is reverted by inhibiting glycolysis and the PPP with 3-bromopyruvate (BrPA) and 6-amino nicotinamide (6AN), respectively [78].

Altered lipid metabolism is also associated with cancer progression. Since cancer cells use lipids as signaling molecules as well as building blocks or an energy source, altered lipid metabolism is observed in the pathogenesis of cancer [79]. Pitroda et al. proposed a 38-gene set, designated as MLMS (MUC1-induced lipid metabolism signature), that consists of differentially expressed genes associated with lipid metabolism in MUC1-transformed 3Y1 cells. The MLMS contains genes involved in cholesterol metabolism, lipid transport, and fatty acid synthesis. MLMS overexpression is associated with a poor prognosis in tamoxifen-treated breast cancer patients, suggesting that altered lipid metabolism may induce tamoxifen resistance [80]. MLMS gene expression patterns are correlated with ER-dependent gene expression, as MUC1 binds to the ERE in association with ERα [29].

### 3.4. EMT and Metastasis

Invasion and metastasis are closely related to a poor prognosis in cancer patients. The epithelial–mesenchymal transition (EMT) is the first stage in cancer cell movement, which is represented by a loss of cell polarity. Along with the concurrent phenotypic changes, the molecular mechanisms underlying EMT have been well-studied [81]. Recently, a series of studies has lent support to the role of the mucins in the EMT in breast and pancreatic cancers. Analyses of MUC1-overexpressing cells and knockout mouse models have demonstrated that the EMT process is strongly affected by MUC1 in pancreatic cancer. As an example of this, the EMT is blocked when all tyrosine residues in the MUC1 cytoplasmic domain are substituted for phenylalanine. This MUC1 mutant cannot bind to β-catenin and therefore fails to translocate to the nucleus to promote the transcription of EMT genes [82]. Grover et al. have reported similar findings—i.e., that the tyrosine residues of the MUC1 cytoplasmic domain are important for TGF-β-induced EMT in pancreatic cancer [83]. The direct association of MUC1 to TWIST1 and ZEB1 (zinc-finger E-box-binding homeobox 1) has also been shown to regulate the EMT process in breast cancer when two major immune-related signal pathways are activated—i.e., STAT3 (signal transducer and activator of transcription 3) and NF-κB, respectively [84,85].

MUC16 is also a mediator of EMT in pancreatic cancer, and its knockdown results in a decreased migration of cancer cells in vitro and reduced metastasis in vivo. Indeed, the recently described interaction between MUC16 and FAK is suggested as a mechanism of pancreatic cancer metastasis [86]. Lakshmanan et al. have demonstrated that MUC16 is expressed in the metastatic lymph nodes of lung cancer patients. A MUC16 knockdown also markedly decreases lung cancer cell migration via JAK2/STAT3/GR (glucocorticoid receptor)-mediated TSPYL5 (testis-specific protein Y-encoded-like 5) downregulation [72].

### 3.5. Avoiding Immune Surveillance

The host immune system continuously eliminates newly transformed cancerous cells by recognizing tumor-specific antigens or cellular stress-induced markers [87]. This process, referred to as “immune surveillance”, is a major hurdle to be overcome by cancer cells for their propagation [88]. Since mucins expressed in normal epithelial tracts have an important role in mucosal immunity against bacterial infection, cancer-associated mucins have been thought to modulate cancer immunity. Mucins engage several strategies to avoid host immunity, including (1) blocking the interaction between immune cells and cancer cells, (2) modulating immune cell signaling via co-stimulatory or co-inhibitory molecules, and (3) regulating proinflammatory cytokine production. Because of the large and glycosylated structure of their extracellular region, mucin proteins have an inhibitory role against cell–cell interactions [89,90].

**Figure 3:**
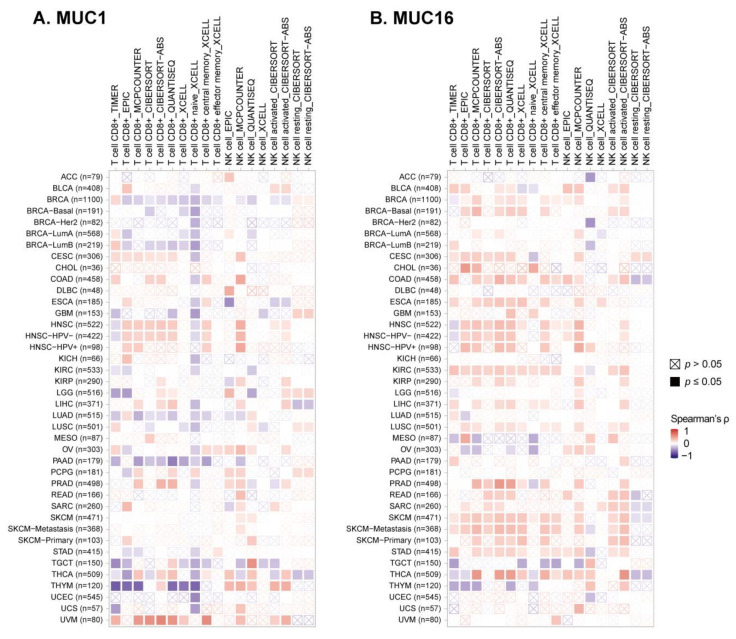
Correlation between mucin expression and tumor-infiltrating T and NK cells. Tumor infiltration analysis of CD8+ T cells and NK cells was conducted using the TIMER2.0 web portal [91]. The row names in the heatmap represent the TCGA tumor types and number of samples analyzed. Various deconvolution methods were applied to the prediction of tumor-infiltrating immune cells using TCGA bulk RNAseq data. The deconvolution methods are indicated by the column names, along with the type of lymphocyte, i.e., TIMER [92], EPIC [93], MCP-counter [94], CIBERSORT [95], quanTIseq [96] and xCell [97].

Immune cell infiltration analysis of TCGA samples has indicated a strong negative correlation between mucin mRNA expression and cytotoxic lymphocyte infiltration of a tumor (Figure 2 and Figure 3) [91]. The infiltration of CD8+ T cells was indicated to be significantly lower in MUC1-high tumors (BRCA, GBM, LGG, PAAD, THYM, and UCEC) and MUC16-high ovarian cancer, which was assessed by several prediction algorithms. Low NK cell infiltration was also predicted by MUC1-high BRCA, GBM, LGG, and UCEC, but this correlation was found to be relatively weaker than that for CD8+ T cell infiltration (Figure 3). Although the mechanism of reduced T or NK infiltration of mucin-high tumors is not yet fully elucidated, several studies have reported immune suppression mechanisms that support these aforementioned results.

Overexpressed MUC1 and MUC4 on the surfaces of cancer cells provide steric hindrance for the conjugation between cancer cells and cytotoxic lymphocytes, resulting in a decreased cancer cell lysis [98,99]. Glycosylated MUC1 on cancer cells directly binds to selectin or siglec family proteins expressed on immune cells including macrophages and suppresses their functions [100,101,102]. Furthermore, MUC1 plays as an immune checkpoint molecule by binding to intercellular adhesion molecule 1 (ICAM-1) on T cells and inhibiting their functions [103,104]. Cancer-associated MUC1 inhibits dendritic cell (DC) maturation and promotes IL-10^high^IL-12^low^ regulatory DC differentiation, which enables tumors to escape immune surveillance [105,106]. MUC1 is also expressed on DCs that contribute to the suppression of immune responses. In MUC1-deficient mice, DCs showed a more activated phenotype with higher expression of co-stimulatory molecules, including CD40, CD80, and CD86, leading to an augmented CD4+ T cell activation [107]. The ovarian cancer antigen MUC16 (CA125) is known to interact with the immune suppressive molecule galectin-1 and with mesothelin on leukocytes [108,109]. Ovarian cancer cell-derived MUC16 induces an attenuated cytotoxic activity of human NK cells with phenotypic alterations [110,111]. MUC1 also plays an intrinsic role in cancer cell immune evasion through its cytoplasmic domain. MUC1 upregulates programmed death-ligand 1 (PD-L1) expression in non-small cell lung cancer (NSCLC), and this is reversed by the MUC1 cytoplasmic domain inhibitor GO-203. The p65/ZEB1 pathway that regulates the transcription of PD-L1, as well as TLR9, IFN-γ, MCP-1 (monocyte chemoattractant protein-1), and GM-CSF (granulocyte-macrophage colony-stimulating factor) in cancer cells, is activated by MUC1 [112]. The similar mechanism of PD-L1 upregulation by MUC1 was reported in triple-negative breast cancer (TNBC) [113]. Proinflammatory cytokines are important for boosting the immune response to cancer cells. Reciprocally, these cytokines also stimulate mucin overexpression in various cancer cells. Interleukin-6 (IL-6) and IFN-γ activate STAT3 and STAT1 proteins, which bind to the MUC1 promoter region to enhance gene transcription in breast cancer cells [114]. TNF-α and IFN-γ increase MUC16 expression in breast, endometrial, and ovarian cancers via NF-κB-mediated transcription regulation [115]. Conversely, MUC1 promotes the expression of proinflammatory cytokines such as IL-6 and TNF-α by binding to their promoter regions, resulting in a feedback loop that promotes chronic inflammation in the malignant microenvironment [116].

## 4. Targeting Transmembrane Mucins for Cancer Treatment

**Figure 4:**
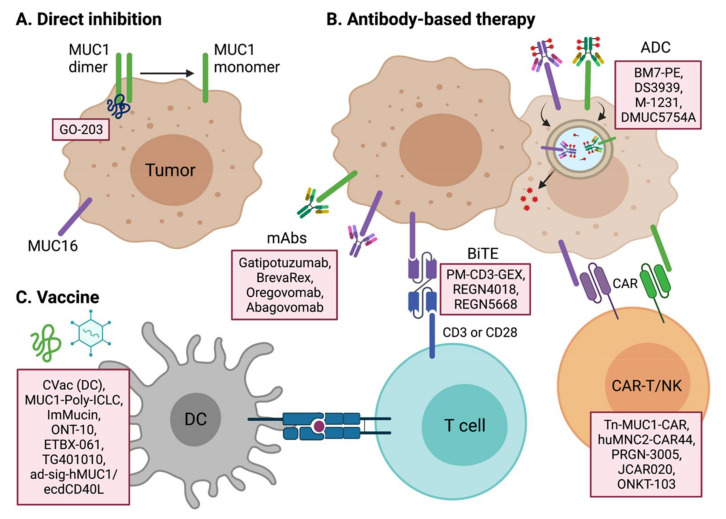
Anti-cancer therapeutic candidates that target MUC1 and MUC16. The aberrant expression of MUC1 and MUC16 in tumors provides potential strategies for targeting these molecules to kill cancer cells. (**A**) The direct MUC1 inhibitor GO-203 is a cell-permeable peptide that binds and blocks MUC1-C. (**B**) The extracellular domain of mucin in cancer cells is a potential target for monoclonal antibody-based therapies. ADCs dump toxins into cancer cells through the endocytosis of MUC1- and MUC16-binding antibodies. BiTE can recruit CD3+ or CD28+ cytotoxic T lymphocytes to MUC1- or MUC16-overexpressing cancer cells. CAR-T or NK cells directly kill cancer cells by recognizing MUC1 and MUC16 antigens. (**C**) Cancer vaccines elicit an active immune response by stimulating antigen-presenting cells (e.g., dendritic cells) against mucin protein antigens.

Many studies have demonstrated that the mucin family of proteins are promising targets for cancer therapeutics. Due to their roles in cancer signal transduction pathways, the signaling pathways of transmembrane mucins may have particular potential in anti-tumor therapy research. The extracellular domain of membrane-bound mucins can also be a good target for antibody-mediated therapies such as neutralizing antibodies, chimeric antigen receptors (CARs), bi-specific T-cell engagers (BiTEs), and antibody–drug conjugates (ADCs). The cancer-specific expression of certain mucin proteins also suggests the possibility of developing a mucin antigen-based cancer vaccine [117]. We describe below the current attempts at developing mucin-targeted cancer therapeutics (Figure 4).

### 4.1. Therapeutic Targeting of MUC1

MUC1 therapeutic candidates are under development for a variety of cancer types, including both solid and blood cancers (Table 1). The absence of an enzymatic pocket inside the MUC1 protein prevents its targeting by a small molecule inhibitor, but peptide inhibitors and RNA aptamers may be viable options for direct-binding inhibitors of MUC1. GO-203 is a cell-penetrating peptide inhibitor of MUC1 dimerization through its direct binding to the CQCRRK region of the MUC1 cytoplasmic domain [118]. The cytoplasmic domain of MUC1 binds a number of key oncogenic proteins, and a block of the dimerization of MUC1 could have anti-tumor effects through a variety of mechanisms, depending on the cell type. Since AKT-S6K1-eIF4A is one of the main pathways altered by MUC1, GO-203 has anti-tumor potency by blocking the AKT pathway in multiple tumor types, such as colon, esophageal, bladder, and breast [119,120,121,122]. GO-203 also shows potential in combination with standard chemotherapies in chemo-resistant cancer cells or hard-to-treat cancer types [121,122]. In TNBC, GO-203 combined with the PARP (poly (ADP-ribose) polymerase) inhibitor olaparib shows anti-cancer potency by blocking MUC1-C-induced epigenetic reprogramming and activating the DNA damage response [123]. In KRAS mutant lung adenocarcinoma, GO-203 suppresses MUC1-induced MYC transcription synergically when combined with the JQ-1 BET inhibitor [124]. GO-203 also shows synergism with lenalidomide and bortezomib against drug-resistant multiple myeloma by regulating TCF4/β-catenin and ER/oxidative stress mechanisms, respectively [125,126]. GO-203 further provides anti-cancer effects against FLT3-mutant leukemia and T cell lymphoma [127,128]. Moreover, in association with the tumor immune microenvironment, GO-203 is known to suppress PD-L1 and induce IFN-γ in NSCLC [129].

Selective RNA aptamer binding to the extracellular domain of MUC1 is another strategy for targeting MUC1-high cancer cells. Perepelyuk et al. have previously designed MUC1-aptamer-hybrid nanoparticles to deliver anti-tumor microRNAs into MUC1-overexpressing cancer cells. These miRNA-29b-loaded hybrid nanoparticles (MAFMILHNs) show anti-tumor effects in a lung cancer mouse model by downregulating DNMT3B (DNA methyltransferase 3 beta), a direct target of the miRNA payload [130]. Furthermore, using a dual payload strategy, geistein-miRNA-29b-biconjugate hybrid nanoparticles (GMLHNs) showed a greater potency than a single payload nanoparticle in a mouse lung cancer model by targeting AKT, PI3K, DNMT3B, and MCL-1 (myeloid cell leukemia-1) [131].

Recent advances in antibody technology have led to a variety of antibody-based therapeutics, such as ADC, BiTE, and CAR therapies, as well as neutralizing therapeutic antibody approaches. BM7-PE and M-1231 are the leading candidates for MUC1 ADCs in present clinical trials. BM7-PE, developed at Oslo University Hospital, comprises anti-MUC1 antibody BM7, conjugated to pseudomonas exotoxin A (PE). In a preclinical study, BM7-PE has shown anti-metastatic effects and promoted long-term survival in a breast cancer nude rat model [132]. BM7-PE is now in a phase 1/2 clinical trial for metastatic colorectal cancer (NCT04550897). M-1231 is a bispecific antibody–drug conjugate targeting the epidermal growth factor receptor (EGFR) and MUC1, and it is now in a phase 1 clinical trial for various metastatic solid tumors. Pab-001 is the first-in-class therapeutic antibody to target OT-MUC1 (onco-tethered MUC1). The highly glycosylated region of transmembrane MUC1 is prone to cleavage by extracellular matrix proteases. The cleaved MUC1-N subunit is released into the blood, thereby sequestering the anti-MUC1 antibody that recognizes the shed MUC1-N domain. Pab-001 targets the extracellular portion of the cleaved MUC1-C subunit to overcome this drawback [133,134]. Pab-001-MMAE ADC has shown promising results against TNBC and other cancers in various preclinical settings. DS-3939 is a PankoMab-GEX (gatipotuzumab) ADC [135], targeting a tumor-specific mucin carbohydrate–protein epitope (TA-MUC1). Bispecific antibodies using PankoMab are under development. PM-CD3-GEX is a BiTE (bispecific T cell engager), which recruits anti-tumor CD3^+^ T cells to MUC1-expressing cancer cells. PM-IL15-GEX is an immunocytokine that combines interleukin-15 with PankoMab-GEX to stimulate anti-tumorigenic NK or T cells. PM-PDL-GEX is a trifunctional antibody targeting MUC1, PD-L1, and FcγR. PD-L1 inhibition and FcγR activation act as an immunostimulant for anti-tumor leukocytes.

After remarkable successes against B cell lymphoma and multiple myeloma, chimeric antigen receptor (CAR) technology is seeking new target molecules for the expansion of its application to solid tumors. Since MUC1 is such a target candidate due to its aberrant expression in various solid tumors, several CAR therapies targeting MUC1 antigen are now under development. It must be noted however that the basal expression of MUC1 in normal tissues can induce significant adverse effects (Figure 2). This has led to new strategies in anti-MUC1 CAR therapies to ensure its safety and efficacy. We below describe recent advances in this regard.

Tn-MUC1 CAR developed by Tmunity Therapeutics is a leading MUC1 CAR-T cell therapy that is currently under phase 1 clinical trial (NCT04025216). Because Tn (GalNAcα1-O-Ser/Thr) is the most prevalent abnormal glycoform found in cancer tissues, the Tn glycoform of MUC1 (Tn-MUC1) is a promising target for CAR therapy. Tn-MUC1 CAR-T has shown anti-tumor potency against T cell lymphomas and pancreatic tumors in a target-specific manner [136]. The MUC-1 pCAR developed by Leucid Bio is a parallel CAR (pCAR) platform that introduces two chimeric antigen receptors side-by-side with different antigen-binding domains and with co-stimulatory domains or cytokine-stimulatory receptors, respectively (WO2020183158). This combination of dual receptors is expected to give T cells more specificity against MUC1-positive tumors and more efficacy than standard CAR-Ts, which have low potency against solid tumors. huMNC2-CAR44 T cells produced by Minerva Biotechnologies Corp are harboring scFv against a cleaved form of MUC1 present on solid cancer cells. huMNC2-CAR44 is in phase 1 clinical trials (NCT04020575) for breast, ovarian, pancreatic, and lung cancer, which are highly MUC1*-positive tumor types. NK cells are also considered as good hosts for CAR therapy. ONKT-103 is a MUC1 targeting CAR-NK cell therapy developed by ONK Therapeutics. ONKT-103 maximizes anti-tumor activity by introducing a DR5-TRAIL variant death receptor signaling pathway. TRAIL in NK cells stimulates the DR5 death receptor of cancer cells and leads to FADD-caspase-mediated apoptosis. ONKT-103 is currently at a preclinical stage and is being tested in the targeting of ovarian, breast, and lung cancers.

### 4.2. Therapeutic Targeting of MUC16 and Other Mucins

Along with MUC1, other membrane-bound mucins have also been considered as potential targets for anti-cancer treatment. We summarize below the various attempts made at targeting MUC16 and other mucins (Table 2).

MUC16 is approved by the FDA for its diagnostic usage [137]. Targeting MUC16 for cancer therapeutics is expected to improve the poor prognosis of ovarian cancer. Oregovomab (OvaRex) is the first monoclonal antibody drug investigated in clinical trials. Oregovomab binds the glycosylated region of MUC16 with high affinity (1.16 × 10^10^/M) and induces indirect immune responses via an anti-idiotype antibody induction cascade [138]. Oregovomab (Ab_1_) induces anti-oregovomab antibodies (anti-idiotype antibodies; Ab_2_), which in turn induces anti-anti-idiotype antibodies (Ab_3_). Ab_3_ antibodies recognize the original MUC16 antigen, resulting in immune cell-mediated killing of MUC16-expressing tumor cells. Various clinical trials of this agent have been conducted in different settings for ovarian cancers [139]. Oregovomab has shown very promising results in a phase 2 trial in combination with carboplatin and paclitaxel (CP), as compared with CP only, for 97 patients with stage III/IV ovarian cancer. The progression-free survival (PFS) outcome was 41.8 months for CP plus oregovomab vs. 12.2 for CP only (*p* = 0.0027, HR = 0.46, 95% CI = 0.28–0.7) [140]. The co-administration of CP with oregovomab resulted in an increase in MUC16-specific IFN-γ^+^ CD8^+^ T lymphocytes in the peripheral blood, demonstrating the activation of an immune response to oregovomab [141]. However, despite encouraging results from a combination study with standard chemotherapies, oregovomab monotherapy did not show a clinical benefit in phase 2 and phase 3 clinical trials [142,143]. Another phase 3 clinical trial (NCT04498117) of oregovomab is ongoing for newly diagnosed ovarian cancer patients in conjunction with carboplatin and paclitaxel chemotherapy.

Abagovomab is an anti-idiotype antibody (Ab_2_), generated against the anti-MUC16 antibody OC125 (Ab_1_). Abagovomab induces a specific Ab_3_ response, which in turn activates a cellular cytotoxic response against MUC16-expressing cancer cells. As an ovarian cancer vaccine for maintenance therapy, abagovomab has shown very promising results in terms of an immune response and overall survival (OS) improvements (median OS 23.5 vs. 4.9 months; *p* < 0.001) in a phase 1b/2 trial [144]. However, a multicenter phase 3 MIMOSA study of abagovomab involving 888 patients (NCT00418574) failed to confirm these clinical benefits (HR for RFS = 1.099; *p* = 0.301, HR for OS = 1.150; *p* = 0.322) [145]. Subsequent analysis of the MIMOSA study findings indicated that abagovomab does not augment MUC16-specific cytotoxic T lymphocytes (CTLs) [146]. A high level of MUC16-specific CTLs was found to be associated with a good prognosis, regardless of abagovomab treatment. Further analysis has suggested that the proportion of IFN-γ^+^ CD8^+^ T cells is a factor determining the clinical benefits of abagovomab and could therefore be a predictive biomarker for this drug [147].

DMUC5754A (RG-7458, sofituzumab vedotin) is an ADC that comprises the humanized anti-MUC16 antibody conjugated to a potent anti-mitotic agent, monomethyl auristatin E (MMAE). A phase 1 study of DMUC5754A was performed for patients with platinum-resistant ovarian cancer (OC) and unresectable pancreatic cancer (PC). Despite the safe profile of DMUC5754A, the response rate was only 17% (5/29; 1 CR; 4 PRs) for the OC cases, with neither CR nor PR observed for any of the PC patients [148]. Regeneron is currently developing MUC16 BiTEs that co-target MUC16-positive cancer and T cells. REGN4018 (MUC16/CD3 BiTE) shows MUC16-dependent anti-tumor potency and good tolerability in both murine and monkey models [149]. REGN4018 is now under phase 2 clinical trials alone and in combination with the PD-1 antibody cemiplimab or with REGN5668 (MUC16/CD28 BiTE) for recurrent ovarian cancer patients (NCT03564340, NCT04590326). JCAR-020, developed by Juno/Celgene/Bristol-Myers Squibb, is a MUC16 CAR-T cell therapy that harbors an interleukin-12 receptor agonist. JCAR-020 is currently under a phase 1 clinical trial (NCT02498912).

In addition to MUC1 and MUC16, other transmembrane mucins have been assessed as potential cancer targets. The aberrant expression and pathogenesis of MUC13 in pancreatic cancer leads to the development of anti-MUC13 antibodies that can be used for diagnostic and therapeutic purposes [150,151,152]. Amgen is developing a BiTE targeting CD3 and MUC17 for the treatment of gastric and esophageal cancer, and this is now in a phase 1 trial (WO2019133961A1, NCT04117958).

### 4.3. Tumor Vaccines

Therapeutic cancer vaccines are designed to activate a host’s immune system to eradicate cancer cells. The host immune system not only generates antibodies that recognize a specific cancer antigen but also induces a CTL-mediated tumor cell killing. Along with mucin-targeting passive immunotherapies such as the administration of a therapeutic antibody or engineered CTLs, vaccination with mucin antigens has also been vigorously attempted for treating various solid tumors.

CVac are autologous monocyte-derived DCs primed with a mannosylated MUC1 protein. Two phase 2 clinical studies have now been conducted with these cells: one for advanced OC patients with progressive disease after standard chemotherapy [153], and one for maintenance therapy after clinical remission in OC patients [154]. The CVac DC vaccine was found to have adequate safety with minimal adverse effects but failed to increase the PFS compared with standard chemotherapy alone. However, in a sub-group analysis that divided participants into first (CR1) and second clinical remission (CR2) groups, CVac produced a promising improvement in the PFS (HR = 0.32) and OS (HR = 0.17) in the CR2 group. This result however was from a small-sized randomized trial (n = 10 for each group), and a phase 3 clinical trial with a large cohort will be needed to verify this finding.

ImMucin is a 21-mer peptide vaccine comprising the signal peptide domain of the MUC1 protein that binds to various MHC class I and class II alleles [155]. A phase 1/2 study of ImMucin for multiple myeloma with co-administration of GM-CSF demonstrated a safe tolerability of this vaccination, the successful induction of a vaccine-mediated cellular and humoral immune response, and clinical disease control in 11/15 patients (duration: 17.5–41.3 months after study completion) [156].

Dr. Finn and colleagues have also designed a peptide sequence from MUC1 as a tumor vaccine. Direct administration of a 100-mer clinical grade peptide (5 repeats of 20-mer peptide) with adjuvants has shown the tolerability and immunogenicity of this vaccine in phase 1 and phase 2 clinical trials for pancreatic cancer and colon cancer patients [157,158]. This peptide has also been exploited as a MUC1 peptide pulsed autologous DC vaccine for patients with pancreatic and biliary tumors after resection of their primary tumors. In that particular clinical study, 4/12 patients survived without recurrence, with a median survival of 26 months (range, 13–69 months) [159].

ONT-10 is a liposome therapeutic vaccine consisting of two repeats of a 20-mer synthetic glycopeptide from MUC1 combined with pentaerythritol lipid A (PET Lipid A), a TLR4 agonist. A preclinical study of ONT-10 indicated an induction of a cellular and humoral immune response to MUC1 and anti-tumor effects in syngenic B16-MUC1 and MC38-MUC1 models [160]. A phase 1 study of 28 advanced solid cancer patients demonstrated that ONT-10 is safe and well-tolerated, but neither CR nor PR was observed [161]. Recently, a phase 1b study of ONT-10 in combination with varlilumab (anti-CD27 agonistic antibody) was performed in advanced ovarian and breast cancer patients (NCT02270372). Emepepimut-S (also known as Tecemotide or L-BLP25) is another developed peptide vaccine for MUC1. However, in a phase 3 study in NSCLC patients, no significant difference in OS was observed [162].

ETBX-061 is a therapeutic adenovirus vaccine targeting the MUC1 protein. Considering the heterogenetic nature of solid tumors, ETBX-061 has been studied in combination with other vaccines or therapeutic agents in clinical trials. A triple (CEA/MUC1/Brachyury) vaccine combination regimen was studied in a phase 1 clinical trial for advanced cancer patients that confirmed antigen-specific T cell generation and disease control (60% SD and 40% PD) [163]. Other clinical studies with different regimens have also been reported (Table 3). TG4010 is a modified vaccinia Ankara (MVA) expressing MUC1 and interleukin-2. In a phase 2b/3 trial for advanced NSCLC, TG4010 plus chemotherapy produced a significant improvement in the PFS relative to a placebo plus chemotherapy, but the survival benefit was marginal only (5.9 vs. 5.1 months) [164]. MicroVAC LLC is developing an ad-sig-hMUC1/ecdCD40L vaccine in which a fusion protein of MUC1 (TAA; tumor-associated antigen) is combined with the extracellular domain (ECD) of the CD40 ligand (CD40L) to boost DC activation and promote T and B cell expansion [165]. A small cohort phase 1 study of this vaccine has demonstrated that it is safe and has encouraging anti-tumor activity [166]. A phase 1 clinical study is now ongoing with a larger number of patients. Other mucin-targeting vaccines under development are summarized in Table 3.

## 5. Conclusions and Perspectives

Transmembrane mucins have important functions in maintaining mucosal structure and physiological homeostasis. Mucins are heavily glycosylated proteins that overexpress in different types of cancers. Many efforts have been continued to find new therapeutic strategies for exploiting the overexpression and aberrant glycosylation of some transmembrane mucins. Many therapeutic agents targeting mucins are under different stages of clinical trial for several cancers. These agents include antibody-based therapeutics, small molecule inhibitors, vaccines, and cell therapy. A better understanding of mucin glycoproteins in terms of shedding mechanism, aberrant glycosylation, possible splice variants, oncogenic signaling cascades, and interacting binding partners would be required to develop more effective mucin-based therapeutic strategies.

## Figures and Tables

**Table 1:** Current therapeutic candidates that target MUC1 protein.

Drug Name	Company	Drug Type	Indication	Development Status	Identifiers or References	Recruitment Status	Start Date
Anti-mucin-1 antibodies	University of California Davis	scFv antibody	Cancer				
Mucin-1 inhibitor	Quest PharmaTech	IgE monoclonal antibody	Cancer	Preclinical			
MUC1 inhibitors	Minerva Biotechnologies Corp.	Humanized single chain antibody	Cancer	Discovery			
Yttrium civatuzumab tetraxetan	Garden State Cancer Center	Radio-labeled antibody	Cancer				
Pab-001	Peptron	ADC	Cancer	Preclinical			
DS-3939	Daiichi Sankyo	ADC	Cancer	Preclinical			
SPmAb	Vaxil BioTherapeutics	Monoclonal antibody	Cancer	Preclinical			
BrevaRex	Quest PharmaTech	Anti-idiotype mAb	Multiple Myeloma, Cancer	Phase 2			
BM7-PE	Oslo University Hospital	ADC	Metastatic colorectal cancer	Phase 2	NCT04550897	Recruiting	31 August 2020
Gatipotuzumab (PankoMab-GEX)	Glycotope GmbH	IgG1 antibody	Advanced ovarian cancer	Phase 2	NCT01899599	Completed	September 2013
PM-CD3-GEX	Glycotope GmbH	BiTE	NSCLC, Ovary tumor	Preclinical			
PM-IL15-GEX	Glycotope GmbH	Antibody fused to IL-15	Solid tumor	Preclinical			
PM-PDL-GEX	Glycotope GmbH	Trifunctional antibody (MUC1, PD-L1, and FcγR)	Ovary tumor	Preclinical			
HuMab-Tn	MabVax Therapeutics Holdings	Monoclonal antibody	Cancer	Discovery			
M-1231	EMD Serono; Sutro Biopharma	ADC	Metastatic cancer	Phase 1	NCT04695847	Recruiting	13 January 2021
Anti-CD3/anti-MUC1 BiTE CIK cell therapy	Benhealth Biopharmaceutical (Shenzhen)	BiTE; Cell therapy	Metastatic cancer	Phase 2	NCT03146637	Recruiting	1 May 2017
GO-203-2C	Genus Oncology LLC	Peptide	R/R AML	Phase 2	NCT02204085	Active, not recruiting	September 2014
GO-203-2C	Genus Oncology LLC	Peptide	Solid tumor	Phase 1	NCT01279603	Completed	19 January 2011
Mucin1-aptamer	Thomas Jefferson University	Aptamer	NSCLC	Preclinical			
rHSP-MUC1 fusion protein	Chengdu Xinlibang Bio-pharmaceutical	Fusion protein	Breast tumor	Phase 1			
P-MUC1C-ALLO1	Poseida Therapeutics	CAR-T	Solid tumor	Preclinical			
P-MUC1C-101	Poseida Therapeutics	CAR-T	Solid tumor	Preclinical			
ICTCAR-052	Innovative Cellular Therapeutics	CAR-T	Breast tumor	Phase 1			
ICTCAR-046	Innovative Cellular Therapeutics	CAR-T	Pancreatic tumor	Phase 1			
ICTCAR-053	Innovative Cellular Therapeutics	CAR-T	Pancreatic tumor	Phase 1			
ICTCAR-043	Innovative Cellular Therapeutics	CAR-T	Breast tumor	Phase 1			
Tn MUC-1 CAR-T	Tmunity Therapeutics	CAR-T	Cancer	Phase 1	NCT04025216	Recruiting	10 October 2019
Anti-MUC1 CAR-T cell therapy (w or w/o) PD-1 knockout T cell	Guangzhou Anjie Biomedical Technology	CAR-T	Metastatic cancer	Phase 2/3	NCT03525782	Recruiting	1 February 2018
MUC-1 pCAR	Leucid Bio	CAR-T	Cancer	Preclinical			
huMNC2-CAR44 T cells	Minerva Biotechnologies Corp.	CAR-T	Cancer	Phase 1	NCT04020575	Recruiting	15 January 2020
Anti-MUC1 CAR-pNK	PersonGen Biomedicine (Suzhou)	CAR-NK	MUC1-positive R/R solid tumor	Phase 2	NCT02839954	Unknown	21 July 2016
Anti-MUC1 CAR-T cell therapy	PersonGen Biomedicine (Suzhou)	CAR-T	MUC1-positive advanced refractory solid tumor	Phase 2	NCT02587689	Unknown	27 October 2015
TAB-28z	OncoTab	CAR-T	Breast tumor	Preclinical			
Anti-MUC-1 CAR-T cell therapy	Baylor College of Medicine	CAR-T	Breast tumor	Preclinical			
ONKT-103	ONK Therapeutics	CAR-NK	Solid tumor	Preclinical			
Anti-MUC1 CAR-T cell therapy + anti-CTLA4 + anti-PD-1 antibodies	Shanghai Cell Therapy Research Institute	CAR-T	MUC1-positive advanced solid tumor	Phase 2	NCT03179007	Unknown	7 June 2017

**Table 2:** Current therapeutic candidates that target MUC16 and other membrane-bound mucin proteins.

Drug Name	Company	Drug Type	Indication	Development Status	Identifiers or References	Recruitment Status	Start Date
Mab-AR-9.6	Quest PharmaTech	Monoclonal antibody	Pancreas tumor	Preclinical			
Oregovomab (OvaRex)	Quest PharmaTech	Monoclonal antibody; Anti-idiotype induction therapy	Cancer	Phase 3	NCT04498117	Recruiting	25 August 2020
RG-7458 (DMUC-5754A)	Genentech	ADC	Ovarian cancer, Pancreatic cancer	Discontinued	NCT01335958	Completed	Day, April 2011
RG-7882 (DMUC-4064A)	Genentech	ADC	Ovarian cancer, Pancreatic cancer	Discontinued	NCT02146313	Completed	22 June 2014
EDO-772P	Mundipharma EDO GmbH	ADC	Ovarian cancer				
Abagovomab	Menarini; CellControl Biomedical	Anti-idiotype mAb	Ovarian cancer	Phase 2/3	NCT00418574	Terminated (Failed)	Day, December 2006
NAV-005	Navrogen	IgG1 Fc fusion protein	Cancer	Discovery			
REGN-4018	Regeneron Pharmaceuticals	BiTE (MUC16/CD3)	Ovarian cancer	Phase 2	NCT03564340	Recruiting	21 May 2018
REGN-5668	Regeneron Pharmaceuticals	BiTE (MUC16/CD28)	Ovarian cancer	Phase 2	NCT04590326	Recruiting	9 December 2020
PRGN-3005	Precigen	CAR-T	Ovarian cancer	Phase 1	NCT03907527	Recruiting	30 April 2019
TC-220	TCR2 Therapeutics	CAR-T	Ovarian cancer	Preclinical			
TC-410	TCR2 Therapeutics	CAR-T	Ovarian cancer	Discovery			
Anti-MUC16 CAR-T/PD-1 scFv	Eureka Therapeutics	CAR-T	Solid tumor	Preclinical			
JCAR-020	Bristol-Myers Squibb	CAR-T	Cancer	Phase 1	NCT02498912	Active, not recruiting	Day, August 2015
Targeting other mucins							
MUC13 antibody	University of Tennessee	Monoclonal antibody	Cancer	Discovery			
AMG-199	Amgen	BiTE (MUC17/CD3)	Stomach cancer, Esophagus cancer	Phase 1	NCT04117958	Recruiting	20 January 2020

**Table 3:** Tumor vaccines that target mucin family proteins.

Drug Name	Company	Drug Type	Indication	Development Status	Identifiers or References	Recruitment Status	Start Date
*Mucin-1 vaccines*							
Cvac	Sydys/Prima BioMed	Autologous DC vaccine	Ovarian cancer, Colorectal cancer, Triple-negative breast cancer	Phase 2	NCT01068509	Completed	July 2010
MUC1-Poly-ICLC	University of Pittsburgh	Peptide vaccine	Colon cancer, NSCLC	Phase 2	NCT00773097	Completed	October 2008
MUC1-DC-CTL	Beijing Doing Biomedical Technology	DC vaccine; T-cell stimulator	Stomach tumor	Preclinical			
ETBX-061	Etubics Corp	Adenovirus vaccine	Colon cancer	Phase 1/2	NCT03563157	Active, not recruiting	25 May 2018
			Advanced cancer	Phase 1	NCT03384316	Completed	31 January 2018
			Hormone refractory prostate cancer	Phase 1	NCT03481816	Completed	24 July 2018
ImMucin	Vaxil BioTherapeutics	Peptide vaccine	Breast cancer, Multiple myeloma, Ovary tumor	Phase 2	NCT01232712	Completed	September 2010
Emepepimut-S (Tecemotide/Stimuvax/L-BLP25)	Merck Serono	Liposome-encapsulated peptide vaccine	Cancer	Phase 3 Discontinued	NCT00409188		
MTI	ViaMune/GeoVax	Peptide vaccine	Cancer	Preclinical			
GEO-CM01	GeoVax	Modified-vaccina Ankara virus-like particles (MVA-VLP)	Cancer	Preclinical			
ONT-10	Cascadian Therapeutics	Glycopeptide vaccine	Cancer	Phase 1b	NCT02270372	Completed	November 2014
TG4010	Transgene SA	MVA	NSCLC	Phase 2/3 (Suspended)	NCT01383148	Terminated	April 2012
TG4010 Ad-sig-hMUC-1/ecdCD40L vaccine	Transgene SA MicroVAX LLC	MVA Adenovirus vaccine	NSCLC Cancer	Phase 2	NCT02823990	Active, not recruiting	14 December 2016
				Phase 1	NCT02140996	Unknown	September 2014
GI-6108	NantCell/Celgene/GlobeImmune	Tarmogen vaccine	Cancer	Preclinical			
*Mucin-16 vaccine*							
OvcaVax	Theralink Technologies	CA-125/IL-2/GM-CSF vaccine	Ovary tumor	Preclinical			

## Data Availability

All data is contained within the article.

## References

[1] Dekker J., Rossen J.W., Buller H.A., Einerhand A.W. (2002). The MUC family: An obituary. Trends Biochem. Sci..

[2] Hollingsworth M.A., Swanson B.J. (2004). Mucins in cancer: Protection and control of the cell surface. Nat. Rev. Cancer.

[3] Bhatia R., Gautam S.K., Cannon A., Thompson C., Hall B.R., Aithal A., Banerjee K., Jain M., Solheim J.C., Kumar S. (2019). Cancer-associated mucins: Role in immune modulation and metastasis. Cancer Metastasis Rev..

[4] Dhanisha S.S., Guruvayoorappan C., Drishya S., Abeesh P. (2018). Mucins: Structural diversity, biosynthesis, its role in pathogenesis and as possible therapeutic targets. Crit. Rev. Oncol. Hematol..

[5] Gendler S.J., Lancaster C.A., Taylor-Papadimitriou J., Duhig T., Peat N., Burchell J., Pemberton L., Lalani E.N., Wilson D. (1990). Molecular cloning and expression of human tumor-associated polymorphic epithelial mucin. J. Biol. Chem..

[6] Batra S.K., Hollingsworth M.A. (1991). Expression of the human mucin gene, Muc 1, in normal tissues and metastatic pancreatic tumors. Int. J. Pancreatol..

[7] Lan M.S., Batra S.K., Qi W.N., Metzgar R.S., Hollingsworth M.A. (1990). Cloning and sequencing of a human pancreatic tumor mucin cDNA. J. Biol. Chem..

[8] Patton S., Gendler S.J., Spicer A.P. (1995). The epithelial mucin, MUC1, of milk, mammary gland and other tissues. Biochim. Biophys. Acta.

[9] Gendler S.J. (2001). MUC1, the renaissance molecule. J. Mammary Gland Biol. Neoplasia.

[10] Kufe D.W. (2009). Mucins in cancer: Function, prognosis and therapy. Nat. Rev. Cancer.

[11] Li Y., Liu D., Chen D., Kharbanda S., Kufe D. (2003). Human DF3/MUC1 carcinoma-associated protein functions as an oncogene. Oncogene.

[12] Li Y., Cozzi P.J. (2007). MUC1 is a promising therapeutic target for prostate cancer therapy. Curr. Cancer Drug. Targets.

[13] Joshi M.D., Ahmad R., Yin L., Raina D., Rajabi H., Bubley G., Kharbanda S., Kufe D. (2009). MUC1 oncoprotein is a druggable target in human prostate cancer cells. Mol. Cancer Ther..

[14] Hu X.F., Yang E., Li J., Xing P.X. (2006). MUC1 cytoplasmic tail: A potential therapeutic target for ovarian carcinoma. Exp. Rev. Anticancer Ther..

[15] Kufe D.W. (2013). MUC1-C oncoprotein as a target in breast cancer: Activation of signaling pathways and therapeutic approaches. Oncogene.

[16] Zeimet A.G., Offner F.A., Muller-Holzner E., Widschwendter M., Abendstein B., Fuith L.C., Daxenbichler G., Marth C. (1998). Peritoneum and tissues of the female reproductive tract as physiological sources of CA-125. Tumour Biol..

[17] Argueso P., Spurr-Michaud S., Russo C.L., Tisdale A., Gipson I.K. (2003). MUC16 mucin is expressed by the human ocular surface epithelia and carries the H185 carbohydrate epitope. Investig. Ophthalmol. Vis. Sci..

[18] Matsuoka Y., Endo K., Kawamura Y., Yoshida T., Saga T., Watanabe Y., Koizumi M., Nakashima T., Konishi J., Yamaguchi N. (1990). Normal bronchial mucus contains high levels of cancer-associated antigens, CA125, CA19-9, and carcinoembryonic antigen. Cancer.

[19] Bast R.C., Xu F.J., Yu Y.H., Barnhill S., Zhang Z., Mills G.B. (1998). CA 125: The past and the future. Int. J. Biol. Markers.

[20] Capstick V., Maclean G.D., Suresh M.R., Bodnar D., Lloyd S., Shepert L., Longenecker B.M., Krantz M. (1991). Clinical evaluation of a new two-site assay for CA125 antigen. Int. J. Biol. Markers.

[21] Singh R., Samant U., Hyland S., Chaudhari P.R., Wels W.S., Bandyopadhyay D. (2007). Target-specific cytotoxic activity of recombinant immunotoxin scFv(MUC1)-ETA on breast carcinoma cells and primary breast tumors. Mol. Cancer Ther..

[22] Singh R., Bandyopadhyay D. (2007). MUC1: A target molecule for cancer therapy. Cancer Biol. Ther..

[23] Hanisch F.G. (2001). O-glycosylation of the mucin type. Biol. Chem..

[24] Carraway K.L., Rossi E.A., Komatsu M., Price-Schiavi S.A., Huang D., Guy P.M., Carvajal M.E., Fregien N., Carraway C.A., Carraway K.L. (1999). An intramembrane modulator of the ErbB2 receptor tyrosine kinase that potentiates neuregulin signaling. J. Biol. Chem..

[25] Al Masri A., Gendler S.J. (2005). Muc1 affects c-Src signaling in PyV MT-induced mammary tumorigenesis. Oncogene.

[26] Kinlough C.L., Poland P.A., Bruns J.B., Harkleroad K.L., Hughey R.P. (2004). MUC1 membrane trafficking is modulated by multiple interactions. J. Biol. Chem..

[27] Li Y., Bharti A., Chen D., Gong J., Kufe D. (1998). Interaction of glycogen synthase kinase 3beta with the DF3/MUC1 carcinoma-associated antigen and beta-catenin. Mol. Cell Biol..

[28] Pandey P., Kharbanda S., Kufe D. (1995). Association of the DF3/MUC1 breast cancer antigen with Grb2 and the Sos/Ras exchange protein. Cancer Res..

[29] Wei X., Xu H., Kufe D. (2006). MUC1 oncoprotein stabilizes and activates estrogen receptor alpha. Mol. Cell.

[30] Yamamoto M., Bharti A., Li Y., Kufe D. (1997). Interaction of the DF3/MUC1 breast carcinoma-associated antigen and beta-catenin in cell adhesion. J. Biol. Chem..

[31] Wei X., Xu H., Kufe D. (2005). Human MUC1 oncoprotein regulates p53-responsive gene transcription in the genotoxic stress response. Cancer Cell.

[32] Oosterkamp H.M., Scheiner L., Stefanova M.C., Lloyd K.O., Finstad C.L. (1997). Comparison of MUC-1 mucin expression in epithelial and non-epithelial cancer cell lines and demonstration of a new short variant form (MUC-1/Z). Int. J. Cancer.

[33] Zrihan-Licht S., Vos H.L., Baruch A., Elroy-Stein O., Sagiv D., Keydar I., Hilkens J., Wreschner D.H. (1994). Characterization and molecular cloning of a novel MUC1 protein, devoid of tandem repeats, expressed in human breast cancer tissue. Eur. J. Biochem..

[34] Zhang L., Vlad A., Milcarek C., Finn O.J. (2013). Human mucin MUC1 RNA undergoes different types of alternative splicing resulting in multiple isoforms. Cancer Immunol. Immunother..

[35] Haridas D., Ponnusamy M.P., Chugh S., Lakshmanan I., Seshacharyulu P., Batra S.K. (2014). MUC16: Molecular analysis and its functional implications in benign and malignant conditions. FASEB J..

[36] Duraisamy S., Ramasamy S., Kharbanda S., Kufe D. (2006). Distinct evolution of the human carcinoma-associated transmembrane mucins, MUC1, MUC4 AND MUC16. Gene.

[37] Blalock T.D., Spurr-Michaud S.J., Tisdale A.S., Heimer S.R., Gilmore M.S., Ramesh V., Gipson I.K. (2007). Functions of MUC16 in corneal epithelial cells. Investig. Ophthalmol. Vis. Sci..

[38] Akita K., Tanaka M., Tanida S., Mori Y., Toda M., Nakada H. (2013). CA125/MUC16 interacts with Src family kinases, and over-expression of its C-terminal fragment in human epithelial cancer cells reduces cell-cell adhesion. Eur. J. Cell Biol..

[39] Tsutsumida H., Goto M., Kitajima S., Kubota I., Hirotsu Y., Wakimoto J., Batra S.K., Imai K., Yonezawa S. (2007). MUC4 expression correlates with poor prognosis in small-sized lung adenocarcinoma. Lung Cancer.

[40] Hanaoka J., Kontani K., Sawai S., Ichinose M., Tezuka N., Inoue S., Fujino S., Ohkubo I. (2001). Analysis of MUC4 mucin expression in lung carcinoma cells and its immunogenicity. Cancer.

[41] Guddo F., Giatromanolaki A., Koukourakis M.I., Reina C., Vignola A.M., Chlouverakis G., Hilkens J., Gatter K.C., Harris A.L., Bonsignore G. (1998). MUC1 (episialin) expression in non-small cell lung cancer is independent of EGFR and c-erbB-2 expression and correlates with poor survival in node positive patients. J. Clin. Pathol..

[42] Rakha E.A., Boyce R.W., Abd El-Rehim D., Kurien T., Green A.R., Paish E.C., Robertson J.F., Ellis I.O. (2005). Expression of mucins (MUC1, MUC2, MUC3, MUC4, MUC5AC and MUC6) and their prognostic significance in human breast cancer. Mod. Pathol..

[43] Hayes D.F., Sekine H., Ohno T., Abe M., Keefe K., Kufe D.W. (1985). Use of a murine monoclonal antibody for detection of circulating plasma DF3 antigen levels in breast cancer patients. J. Clin. Investig..

[44] Apostolopoulos V., McKenzie I.F. (1994). Cellular mucins: Targets for immunotherapy. Crit. Rev. Immunol..

[45] Choudhury A., Moniaux N., Winpenny J.P., Hollingsworth M.A., Aubert J.P., Batra S.K. (2000). Human MUC4 mucin cDNA and its variants in pancreatic carcinoma. J. Biochem..

[46] Balague C., Gambus G., Carrato C., Porchet N., Aubert J.P., Kim Y.S., Real F.X. (1994). Altered expression of MUC2, MUC4, and MUC5 mucin genes in pancreas tissues and cancer cell lines. Gastroenterology.

[47] Andrianifahanana M., Moniaux N., Schmied B.M., Ringel J., Friess H., Hollingsworth M.A., Buchler M.W., Aubert J.P., Batra S.K. (2001). Mucin (MUC) gene expression in human pancreatic adenocarcinoma and chronic pancreatitis: A potential role of MUC4 as a tumor marker of diagnostic significance. Clin. Cancer Res..

[48] Wang R.Q., Fang D.C. (2003). Alterations of MUC1 and MUC3 expression in gastric carcinoma: Relevance to patient clinicopathological features. J. Clin. Pathol..

[49] Utsunomiya T., Yonezawa S., Sakamoto H., Kitamura H., Hokita S., Aiko T., Tanaka S., Irimura T., Kim Y.S., Sato E. (1998). Expression of MUC1 and MUC2 mucins in gastric carcinomas: Its relationship with the prognosis of the patients. Clin. Cancer Res..

[50] Walsh M.D., Young J.P., Leggett B.A., Williams S.H., Jass J.R., McGuckin M.A. (2007). The MUC13 cell surface mucin is highly expressed by human colorectal carcinomas. Hum. Pathol..

[51] Duncan T.J., Watson N.F., Al-Attar A.H., Scholefield J.H., Durrant L.G. (2007). The role of MUC1 and MUC3 in the biology and prognosis of colorectal cancer. World J. Surg. Oncol..

[52] Fritsche H.A., Bast R.C. (1998). CA 125 in ovarian cancer: Advances and controversy. Clin. Chem..

[53] Chauhan S.C., Singh A.P., Ruiz F., Johansson S.L., Jain M., Smith L.M., Moniaux N., Batra S.K. (2006). Aberrant expression of MUC4 in ovarian carcinoma: Diagnostic significance alone and in combination with MUC1 and MUC16 (CA125). Mod. Pathol..

[54] Hanahan D., Weinberg R.A. (2011). Hallmarks of cancer: The next generation. Cell.

[55] Tang Z., Kang B., Li C., Chen T., Zhang Z. (2019). GEPIA2: An enhanced web server for large-scale expression profiling and interactive analysis. Nucleic Acids Res..

[56] Regad T. (2015). Targeting RTK Signaling Pathways in Cancer. Cancers.

[57] Li Y., Ren J., Yu W., Li Q., Kuwahara H., Yin L., Carraway K.L., Kufe D. (2001). The epidermal growth factor receptor regulates interaction of the human DF3/MUC1 carcinoma antigen with c-Src and beta-catenin. J. Biol. Chem..

[58] Pochampalli M.R., el Bejjani R.M., Schroeder J.A. (2007). MUC1 is a novel regulator of ErbB1 receptor trafficking. Oncogene.

[59] Schroeder J.A., Thompson M.C., Gardner M.M., Gendler S.J. (2001). Transgenic MUC1 interacts with epidermal growth factor receptor and correlates with mitogen-activated protein kinase activation in the mouse mammary gland. J. Biol. Chem..

[60] Ren J., Raina D., Chen W., Li G., Huang L., Kufe D. (2006). MUC1 oncoprotein functions in activation of fibroblast growth factor receptor signaling. Mol. Cancer Res..

[61] Huang L., Chen D., Liu D., Yin L., Kharbanda S., Kufe D. (2005). MUC1 oncoprotein blocks glycogen synthase kinase 3beta-mediated phosphorylation and degradation of beta-catenin. Cancer Res..

[62] Jin W., Liao X., Lv Y., Pang Z., Wang Y., Li Q., Liao Y., Ye Q., Chen G., Zhao K. (2017). MUC1 induces acquired chemoresistance by upregulating ABCB1 in EGFR-dependent manner. Cell Death Dis..

[63] Nath S., Daneshvar K., Roy L.D., Grover P., Kidiyoor A., Mosley L., Sahraei M., Mukherjee P. (2013). MUC1 induces drug resistance in pancreatic cancer cells via upregulation of multidrug resistance genes. Oncogenesis.

[64] Ren J., Agata N., Chen D., Li Y., Yu W.H., Huang L., Raina D., Chen W., Kharbanda S., Kufe D. (2004). Human MUC1 carcinoma-associated protein confers resistance to genotoxic anticancer agents. Cancer Cell.

[65] Raina D., Kharbanda S., Kufe D. (2004). The MUC1 oncoprotein activates the anti-apoptotic phosphoinositide 3-kinase/Akt and Bcl-xL pathways in rat 3Y1 fibroblasts. J. Biol. Chem..

[66] Kharbanda S., Ren R., Pandey P., Shafman T.D., Feller S.M., Weichselbaum R.R., Kufe D.W. (1995). Activation of the c-Abl tyrosine kinase in the stress response to DNA-damaging agents. Nature.

[67] Raina D., Ahmad R., Kumar S., Ren J., Yoshida K., Kharbanda S., Kufe D. (2006). MUC1 oncoprotein blocks nuclear targeting of c-Abl in the apoptotic response to DNA damage. EMBO J..

[68] Ahmad R., Raina D., Trivedi V., Ren J., Rajabi H., Kharbanda S., Kufe D. (2007). MUC1 oncoprotein activates the IkappaB kinase beta complex and constitutive NF-kappaB signalling. Nat. Cell Biol..

[69] Yin L., Huang L., Kufe D. (2004). MUC1 oncoprotein activates the FOXO3a transcription factor in a survival response to oxidative stress. J. Biol. Chem..

[70] Agata N., Ahmad R., Kawano T., Raina D., Kharbanda S., Kufe D. (2008). MUC1 oncoprotein blocks death receptor-mediated apoptosis by inhibiting recruitment of caspase-8. Cancer Res..

[71] Boivin M., Lane D., Piche A., Rancourt C. (2009). CA125 (MUC16) tumor antigen selectively modulates the sensitivity of ovarian cancer cells to genotoxic drug-induced apoptosis. Gynecol Oncol..

[72] Lakshmanan I., Salfity S., Seshacharyulu P., Rachagani S., Thomas A., Das S., Majhi P.D., Nimmakayala R.K., Vengoji R., Lele S.M. (2017). MUC16 Regulates TSPYL5 for Lung Cancer Cell Growth and Chemoresistance by Suppressing p53. Clin. Cancer Res..

[73] DeBerardinis R.J., Chandel N.S. (2016). Fundamentals of cancer metabolism. Sci. Adv..

[74] Chaika N.V., Gebregiworgis T., Lewallen M.E., Purohit V., Radhakrishnan P., Liu X., Zhang B., Mehla K., Brown R.B., Caffrey T. (2012). MUC1 mucin stabilizes and activates hypoxia-inducible factor 1 alpha to regulate metabolism in pancreatic cancer. Proc. Natl. Acad. Sci. USA.

[75] Shukla S.K., Gunda V., Abrego J., Haridas D., Mishra A., Souchek J., Chaika N.V., Yu F., Sasson A.R., Lazenby A.J. (2015). MUC16-mediated activation of mTOR and c-Myc reprograms pancreatic cancer metabolism. Oncotarget.

[76] Kosugi M., Ahmad R., Alam M., Uchida Y., Kufe D. (2011). MUC1-C oncoprotein regulates glycolysis and pyruvate kinase M2 activity in cancer cells. PLoS ONE.

[77] Turgeon M.O., Perry N.J.S., Poulogiannis G. (2018). DNA Damage, Repair, and Cancer Metabolism. Front. Oncol..

[78] Gunda V., Souchek J., Abrego J., Shukla S.K., Goode G.D., Vernucci E., Dasgupta A., Chaika N.V., King R.J., Li S. (2017). MUC1-Mediated Metabolic Alterations Regulate Response to Radiotherapy in Pancreatic Cancer. Clin. Cancer Res..

[79] Snaebjornsson M.T., Janaki-Raman S., Schulze A. (2020). Greasing the Wheels of the Cancer Machine: The Role of Lipid Metabolism in Cancer. Cell Metab..

[80] Pitroda S.P., Khodarev N.N., Beckett M.A., Kufe D.W., Weichselbaum R.R. (2009). MUC1-induced alterations in a lipid metabolic gene network predict response of human breast cancers to tamoxifen treatment. Proc. Natl. Acad. Sci. USA.

[81] Ribatti D., Tamma R., Annese T. (2020). Epithelial-Mesenchymal Transition in Cancer: A Historical Overview. Transl. Oncol..

[82] Roy L.D., Sahraei M., Subramani D.B., Besmer D., Nath S., Tinder T.L., Bajaj E., Shanmugam K., Lee Y.Y., Hwang S.I. (2011). MUC1 enhances invasiveness of pancreatic cancer cells by inducing epithelial to mesenchymal transition. Oncogene.

[83] Grover P., Nath S., Nye M.D., Zhou R., Ahmad M., Mukherjee P. (2018). SMAD4-independent activation of TGF-beta signaling by MUC1 in a human pancreatic cancer cell line. Oncotarget.

[84] Rajabi H., Alam M., Takahashi H., Kharbanda A., Guha M., Ahmad R., Kufe D. (2014). MUC1-C oncoprotein activates the ZEB1/miR-200c regulatory loop and epithelial-mesenchymal transition. Oncogene.

[85] Hata T., Rajabi H., Yamamoto M., Jin C., Ahmad R., Zhang Y., Kui L., Li W., Yasumizu Y., Hong D. (2019). Targeting MUC1-C Inhibits TWIST1 Signaling in Triple-Negative Breast Cancer. Mol. Cancer Ther..

[86] Muniyan S., Haridas D., Chugh S., Rachagani S., Lakshmanan I., Gupta S., Seshacharyulu P., Smith L.M., Ponnusamy M.P., Batra S.K. (2016). MUC16 contributes to the metastasis of pancreatic ductal adenocarcinoma through focal adhesion mediated signaling mechanism. Genes Cancer.

[87] Swann J.B., Smyth M.J. (2007). Immune surveillance of tumors. J. Clin. Investig..

[88] Vinay D.S., Ryan E.P., Pawelec G., Talib W.H., Stagg J., Elkord E., Lichtor T., Decker W.K., Whelan R.L., Kumara H. (2015). Immune evasion in cancer: Mechanistic basis and therapeutic strategies. Semin. Cancer Biol..

[89] Wesseling J., van der Valk S.W., Hilkens J. (1996). A mechanism for inhibition of E-cadherin-mediated cell-cell adhesion by the membrane-associated mucin episialin/MUC1. Mol. Biol. Cell.

[90] Zhang K., Baeckstrom D., Brevinge H., Hansson G.C. (1996). Secreted MUC1 mucins lacking their cytoplasmic part and carrying sialyl-Lewis a and x epitopes from a tumor cell line and sera of colon carcinoma patients can inhibit HL-60 leukocyte adhesion to E-selectin-expressing endothelial cells. J. Cell Biochem..

[91] Li T., Fu J., Zeng Z., Cohen D., Li J., Chen Q., Li B., Liu X.S. (2020). TIMER2.0 for analysis of tumor-infiltrating immune cells. Nucleic Acids Res..

[92] Li B., Severson E., Pignon J.C., Zhao H., Li T., Novak J., Jiang P., Shen H., Aster J.C., Rodig S. (2016). Comprehensive analyses of tumor immunity: Implications for cancer immunotherapy. Genome Biol..

[93] Racle J., de Jonge K., Baumgaertner P., Speiser D.E., Gfeller D. (2017). Simultaneous enumeration of cancer and immune cell types from bulk tumor gene expression data. eLife.

[94] Becht E., Giraldo N.A., Lacroix L., Buttard B., Elarouci N., Petitprez F., Selves J., Laurent-Puig P., Sautes-Fridman C., Fridman W.H. (2016). Estimating the population abundance of tissue-infiltrating immune and stromal cell populations using gene expression. Genome Biol..

[95] Newman A.M., Liu C.L., Green M.R., Gentles A.J., Feng W., Xu Y., Hoang C.D., Diehn M., Alizadeh A.A. (2015). Robust enumeration of cell subsets from tissue expression profiles. Nat. Methods.

[96] Finotello F., Mayer C., Plattner C., Laschober G., Rieder D., Hackl H., Krogsdam A., Loncova Z., Posch W., Wilflingseder D. (2019). Molecular and pharmacological modulators of the tumor immune contexture revealed by deconvolution of RNA-seq data. Genome Med..

[97] Aran D., Hu Z., Butte A.J. (2017). xCell: Digitally portraying the tissue cellular heterogeneity landscape. Genome Biol..

[98] Komatsu M., Yee L., Carraway K.L. (1999). Overexpression of sialomucin complex, a rat homologue of MUC4, inhibits tumor killing by lymphokine-activated killer cells. Cancer Res..

[99] Van de Wiel-van Kemenade E., Ligtenberg M.J., de Boer A.J., Buijs F., Vos H.L., Melief C.J., Hilkens J., Figdor C.G. (1993). Episialin (MUC1) inhibits cytotoxic lymphocyte-target cell interaction. J. Immunol..

[100] Kim Y.J., Borsig L., Han H.L., Varki N.M., Varki A. (1999). Distinct selectin ligands on colon carcinoma mucins can mediate pathological interactions among platelets, leukocytes, and endothelium. Am. J. Pathol..

[101] Nath D., Hartnell A., Happerfield L., Miles D.W., Burchell J., Taylor-Papadimitriou J., Crocker P.R. (1999). Macrophage-tumour cell interactions: Identification of MUC1 on breast cancer cells as a potential counter-receptor for the macrophage-restricted receptor, sialoadhesin. Immunology.

[102] Brinkman-Van der Linden E.C., Varki A. (2000). New aspects of siglec binding specificities, including the significance of fucosylation and of the sialyl-Tn epitope. Sialic acid-binding immunoglobulin superfamily lectins. J. Biol. Chem..

[103] Regimbald L.H., Pilarski L.M., Longenecker B.M., Reddish M.A., Zimmermann G., Hugh J.C. (1996). The breast mucin MUCI as a novel adhesion ligand for endothelial intercellular adhesion molecule 1 in breast cancer. Cancer Res..

[104] Agrawal B., Krantz M.J., Reddish M.A., Longenecker B.M. (1998). Cancer-associated MUC1 mucin inhibits human T-cell proliferation, which is reversible by IL-2. Nat. Med..

[105] Rughetti A., Pellicciotta I., Biffoni M., Backstrom M., Link T., Bennet E.P., Clausen H., Noll T., Hansson G.C., Burchell J.M. (2005). Recombinant tumor-associated MUC1 glycoprotein impairs the differentiation and function of dendritic cells. J. Immunol..

[106] Monti P., Leone B.E., Zerbi A., Balzano G., Cainarca S., Sordi V., Pontillo M., Mercalli A., Di Carlo V., Allavena P. (2004). Tumor-derived MUC1 mucins interact with differentiating monocytes and induce IL-10highIL-12low regulatory dendritic cell. J. Immunol..

[107] Williams M.A., Bauer S., Lu W., Guo J., Walter S., Bushnell T.P., Lillehoj E.P., Georas S.N. (2010). Deletion of the mucin-like molecule muc1 enhances dendritic cell activation in response to toll-like receptor ligands. J. Innate Immunol..

[108] Gubbels J.A., Belisle J., Onda M., Rancourt C., Migneault M., Ho M., Bera T.K., Connor J., Sathyanarayana B.K., Lee B. (2006). Mesothelin-MUC16 binding is a high affinity, N-glycan dependent interaction that facilitates peritoneal metastasis of ovarian tumors. Mol. Cancer.

[109] Seelenmeyer C., Wegehingel S., Lechner J., Nickel W. (2003). The cancer antigen CA125 represents a novel counter receptor for galectin-1. J. Cell Sci..

[110] Belisle J.A., Gubbels J.A., Raphael C.A., Migneault M., Rancourt C., Connor J.P., Patankar M.S. (2007). Peritoneal natural killer cells from epithelial ovarian cancer patients show an altered phenotype and bind to the tumour marker MUC16 (CA125). Immunology.

[111] Patankar M.S., Jing Y., Morrison J.C., Belisle J.A., Lattanzio F.A., Deng Y., Wong N.K., Morris H.R., Dell A., Clark G.F. (2005). Potent suppression of natural killer cell response mediated by the ovarian tumor marker CA125. Gynecol. Oncol..

[112] Bouillez A., Rajabi H., Jin C., Samur M., Tagde A., Alam M., Hiraki M., Maeda T., Hu X., Adeegbe D. (2017). MUC1-C integrates PD-L1 induction with repression of immune effectors in non-small-cell lung cancer. Oncogene.

[113] Maeda T., Hiraki M., Jin C., Rajabi H., Tagde A., Alam M., Bouillez A., Hu X., Suzuki Y., Miyo M. (2018). MUC1-C Induces PD-L1 and Immune Evasion in Triple-Negative Breast Cancer. Cancer Res..

[114] Gaemers I.C., Vos H.L., Volders H.H., van der Valk S.W., Hilkens J. (2001). A stat-responsive element in the promoter of the episialin/MUC1 gene is involved in its overexpression in carcinoma cells. J. Biol. Chem..

[115] Morgado M., Sutton M.N., Simmons M., Warren C.R., Lu Z., Constantinou P.E., Liu J., Francis L.L., Conlan R.S., Bast R.C. (2016). Tumor necrosis factor-alpha and interferon-gamma stimulate MUC16 (CA125) expression in breast, endometrial and ovarian cancers through NFkappaB. Oncotarget.

[116] Cascio S., Zhang L., Finn O.J. (2011). MUC1 protein expression in tumor cells regulates transcription of proinflammatory cytokines by forming a complex with nuclear factor-kappaB p65 and binding to cytokine promoters: Importance of extracellular domain. J. Biol. Chem..

[117] Graham R.A., Burchell J.M., Taylor-Papadimitriou J. (1996). The polymorphic epithelial mucin: Potential as an immunogen for a cancer vaccine. Cancer Immunol. Immunother..

[118] Raina D., Agarwal P., Lee J., Bharti A., McKnight C.J., Sharma P., Kharbanda S., Kufe D. (2015). Characterization of the MUC1-C Cytoplasmic Domain as a Cancer Target. PLoS ONE.

[119] Ahmad R., Alam M., Hasegawa M., Uchida Y., Al-Obaid O., Kharbanda S., Kufe D. (2017). Targeting MUC1-C inhibits the AKT-S6K1-elF4A pathway regulating TIGAR translation in colorectal cancer. Mol. Cancer.

[120] GongSun X., Zhao Y., Jiang B., Xin Z., Shi M., Song L., Qin Q., Wang Q., Liu X. (2019). Inhibition of MUC1-C regulates metabolism by AKT pathway in esophageal squamous cell carcinoma. J. Cell Physiol..

[121] Shigeta K., Hasegawa M., Kikuchi E., Yasumizu Y., Kosaka T., Mizuno R., Mikami S., Miyajima A., Kufe D., Oya M. (2020). Role of the MUC1-C oncoprotein in the acquisition of cisplatin resistance by urothelial carcinoma. Cancer Sci..

[122] Raina D., Uchida Y., Kharbanda A., Rajabi H., Panchamoorthy G., Jin C., Kharbanda S., Scaltriti M., Baselga J., Kufe D. (2014). Targeting the MUC1-C oncoprotein downregulates HER2 activation and abrogates trastuzumab resistance in breast cancer cells. Oncogene.

[123] Yamamoto M., Jin C., Hata T., Yasumizu Y., Zhang Y., Hong D., Maeda T., Miyo M., Hiraki M., Suzuki Y. (2019). MUC1-C Integrates Chromatin Remodeling and PARP1 Activity in the DNA Damage Response of Triple-Negative Breast Cancer Cells. Cancer Res..

[124] Bouillez A., Rajabi H., Pitroda S., Jin C., Alam M., Kharbanda A., Tagde A., Wong K.K., Kufe D. (2016). Inhibition of MUC1-C Suppresses MYC Expression and Attenuates Malignant Growth in KRAS Mutant Lung Adenocarcinomas. Cancer Res..

[125] Yin L., Kufe T., Avigan D., Kufe D. (2014). Targeting MUC1-C is synergistic with bortezomib in downregulating TIGAR and inducing ROS-mediated myeloma cell death. Blood.

[126] Yin L., Tagde A., Gali R., Tai Y.T., Hideshima T., Anderson K., Avigan D., Kufe D. (2017). MUC1-C is a target in lenalidomide resistant multiple myeloma. Br. J. Haematol..

[127] Jain S., Washington A., Leaf R.K., Bhargava P., Clark R.A., Kupper T.S., Stroopinsky D., Pyzer A., Cole L., Nahas M. (2017). Decitabine Priming Enhances Mucin 1 Inhibition Mediated Disruption of Redox Homeostasis in Cutaneous T-Cell Lymphoma. Mol. Cancer Ther..

[128] Liu S., Yin L., Stroopinsky D., Rajabi H., Puissant A., Stegmaier K., Avigan D., Kharbanda S., Kufe D., Stone R. (2014). MUC1-C oncoprotein promotes FLT3 receptor activation in acute myeloid leukemia cells. Blood.

[129] Bouillez A., Adeegbe D., Jin C., Hu X., Tagde A., Alam M., Rajabi H., Wong K.K., Kufe D. (2017). MUC1-C promotes the suppressive immune microenvironment in non-small cell lung cancer. Oncoimmunology.

[130] Perepelyuk M., Sacko K., Thangavel K., Shoyele S.A. (2018). Evaluation of MUC1-Aptamer Functionalized Hybrid Nanoparticles for Targeted Delivery of miRNA-29b to Nonsmall Cell Lung Cancer. Mol. Pharm..

[131] Sacko K., Thangavel K., Shoyele S.A. (2019). Codelivery of Genistein and miRNA-29b to A549 Cells Using Aptamer-Hybrid Nanoparticle Bioconjugates. Nanomaterials.

[132] Engebraaten O., Sivam G., Juell S., Fodstad O. (2000). Systemic immunotoxin treatment inhibits formation of human breast cancer metastasis and tumor growth in nude rats. Int. J. Cancer.

[133] Wu G., Kim D., Kim J.N., Park S., Maharjan S., Koh H., Moon K., Lee Y., Kwon H.J. (2018). A Mucin1 C-terminal Subunit-directed Monoclonal Antibody Targets Overexpressed Mucin1 in Breast Cancer. Theranostics.

[134] Wu G., Maharjan S., Kim D., Kim J.N., Park B.K., Koh H., Moon K., Lee Y., Kwon H.J. (2018). A Novel Monoclonal Antibody Targets Mucin1 and Attenuates Growth in Pancreatic Cancer Model. Int. J. Mol. Sci..

[135] Danielczyk A., Stahn R., Faulstich D., Loffler A., Marten A., Karsten U., Goletz S. (2006). PankoMab: A potent new generation anti-tumour MUC1 antibody. Cancer Immunol. Immunother..

[136] Posey A.D., Schwab R.D., Boesteanu A.C., Steentoft C., Mandel U., Engels B., Stone J.D., Madsen T.D., Schreiber K., Haines K.M. (2016). Engineered CAR T Cells Targeting the Cancer-Associated Tn-Glycoform of the Membrane Mucin MUC1 Control Adenocarcinoma. Immunity.

[137] Bottoni P., Scatena R. (2015). The Role of CA 125 as Tumor Marker: Biochemical and Clinical Aspects. Adv. Exp. Med. Biol..

[138] Schultes B.C., Baum R.P., Niesen A., Noujaim A.A., Madiyalakan R. (1998). Anti-idiotype induction therapy: Anti-CA125 antibodies (Ab3) mediated tumor killing in patients treated with Ovarex mAb B43.13 (Ab1). Cancer Immunol. Immunother..

[139] Pietragalla A., Duranti S., Daniele G., Nero C., Ciccarone F., Lorusso D., Fagotti A., Scambia G. (2021). Oregovomab: An investigational agent for the treatment of advanced ovarian cancer. Expert Opin. Investig. Drugs.

[140] Brewer M., Angioli R., Scambia G., Lorusso D., Terranova C., Panici P.B., Raspagliesi F., Scollo P., Plotti F., Ferrandina G. (2020). Front-line chemo-immunotherapy with carboplatin-paclitaxel using oregovomab indirect immunization in advanced ovarian cancer: A randomized phase II study. Gynecol. Oncol..

[141] Battaglia A., Buzzonetti A., Fossati M., Scambia G., Fattorossi A., Madiyalakan M.R., Mahnke Y.D., Nicodemus C. (2020). Translational immune correlates of indirect antibody immunization in a randomized phase II study using scheduled combination therapy with carboplatin/paclitaxel plus oregovomab in ovarian cancer patients. Cancer Immunol. Immunother..

[142] Berek J.S., Taylor P.T., Gordon A., Cunningham M.J., Finkler N., Orr J., Rivkin S., Schultes B.C., Whiteside T.L., Nicodemus C.F. (2004). Randomized, placebo-controlled study of oregovomab for consolidation of clinical remission in patients with advanced ovarian cancer. J. Clin. Oncol..

[143] Berek J., Taylor P., McGuire W., Smith L.M., Schultes B., Nicodemus C.F. (2009). Oregovomab maintenance monoimmunotherapy does not improve outcomes in advanced ovarian cancer. J. Clin. Oncol..

[144] Reinartz S., Kohler S., Schlebusch H., Krista K., Giffels P., Renke K., Huober J., Mobus V., Kreienberg R., DuBois A. (2004). Vaccination of patients with advanced ovarian carcinoma with the anti-idiotype ACA125: Immunological response and survival (phase Ib/II). Clin. Cancer Res..

[145] Sabbatini P., Harter P., Scambia G., Sehouli J., Meier W., Wimberger P., Baumann K.H., Kurzeder C., Schmalfeldt B., Cibula D. (2013). Abagovomab as maintenance therapy in patients with epithelial ovarian cancer: A phase III trial of the AGO OVAR, COGI, GINECO, and GEICO--the MIMOSA study. J. Clin. Oncol..

[146] Buzzonetti A., Fossati M., Catzola V., Scambia G., Fattorossi A., Battaglia A. (2014). Immunological response induced by abagovomab as a maintenance therapy in patients with epithelial ovarian cancer: Relationship with survival-a substudy of the MIMOSA trial. Cancer Immunol. Immunother..

[147] Battaglia A., Fossati M., Buzzonetti A., Scambia G., Fattorossi A. (2017). A robust immune system conditions the response to abagovomab (anti-idiotypic monoclonal antibody mimicking the CA125 protein) vaccination in ovarian cancer patients. Immunol. Lett..

[148] Liu J.F., Moore K.N., Birrer M.J., Berlin S., Matulonis U.A., Infante J.R., Wolpin B., Poon K.A., Firestein R., Xu J. (2016). Phase I study of safety and pharmacokinetics of the anti-MUC16 antibody-drug conjugate DMUC5754A in patients with platinum-resistant ovarian cancer or unresectable pancreatic cancer. Ann. Oncol..

[149] Crawford A., Haber L., Kelly M.P., Vazzana K., Canova L., Ram P., Pawashe A., Finney J., Jalal S., Chiu D. (2019). A Mucin 16 bispecific T cell-engaging antibody for the treatment of ovarian cancer. Sci. Transl. Med..

[150] Khan S., Zafar N., Khan S.S., Setua S., Behrman S.W., Stiles Z.E., Yallapu M.M., Sahay P., Ghimire H., Ise T. (2018). Clinical significance of MUC13 in pancreatic ductal adenocarcinoma. HPB.

[151] Nishii Y., Yamaguchi M., Kimura Y., Hasegawa T., Aburatani H., Uchida H., Hirata K., Sakuma Y. (2015). A newly developed anti-Mucin 13 monoclonal antibody targets pancreatic ductal adenocarcinoma cells. Int. J. Oncol..

[152] Chauhan S.C., Ebeling M.C., Maher D.M., Koch M.D., Watanabe A., Aburatani H., Lio Y., Jaggi M. (2012). MUC13 mucin augments pancreatic tumorigenesis. Mol. Cancer Ther..

[153] Mitchell P.L., Quinn M.A., Grant P.T., Allen D.G., Jobling T.W., White S.C., Zhao A., Karanikas V., Vaughan H., Pietersz G. (2014). A phase 2, single-arm study of an autologous dendritic cell treatment against mucin 1 in patients with advanced epithelial ovarian cancer. J. Immunother. Cancer.

[154] Gray H.J., Benigno B., Berek J., Chang J., Mason J., Mileshkin L., Mitchell P., Moradi M., Recio F.O., Michener C.M. (2016). Progression-free and overall survival in ovarian cancer patients treated with CVac, a mucin 1 dendritic cell therapy in a randomized phase 2 trial. J. Immunother. Cancer.

[155] Kovjazin R., Volovitz I., Kundel Y., Rosenbaum E., Medalia G., Horn G., Smorodinsky N.I., Brenner B., Carmon L. (2011). ImMucin: A novel therapeutic vaccine with promiscuous MHC binding for the treatment of MUC1-expressing tumors. Vaccine.

[156] Carmon L., Avivi I., Kovjazin R., Zuckerman T., Dray L., Gatt M.E., Or R., Shapira M.Y. (2015). Phase I/II study exploring ImMucin, a pan-major histocompatibility complex, anti-MUC1 signal peptide vaccine, in multiple myeloma patients. Br. J. Haematol..

[157] Kimura T., McKolanis J.R., Dzubinski L.A., Islam K., Potter D.M., Salazar A.M., Schoen R.E., Finn O.J. (2013). MUC1 vaccine for individuals with advanced adenoma of the colon: A cancer immunoprevention feasibility study. Cancer Prev. Res..

[158] Ramanathan R.K., Lee K.M., McKolanis J., Hitbold E., Schraut W., Moser A.J., Warnick E., Whiteside T., Osborne J., Kim H. (2005). Phase I study of a MUC1 vaccine composed of different doses of MUC1 peptide with SB-AS2 adjuvant in resected and locally advanced pancreatic cancer. Cancer Immunol. Immunother..

[159] Lepisto A.J., Moser A.J., Zeh H., Lee K., Bartlett D., McKolanis J.R., Geller B.A., Schmotzer A., Potter D.P., Whiteside T. (2008). A phase I/II study of a MUC1 peptide pulsed autologous dendritic cell vaccine as adjuvant therapy in patients with resected pancreatic and biliary tumors. Cancer Ther..

[160] Pestano L.A., Christian B., Koppenol S., Millard J., Christianson G., Klucher K., Rosler R., Peterson S.R. (2011). Abstract 762: ONT-10, a liposomal vaccine targeting hypoglycosylated MUC1, induces a potent cellular and humoral response and suppresses the growth of MUC1 expressing tumors. Cancer Res..

[161] Nemunaitis J., Bedell C., Klucher K., Vo A., Whiting S. (2013). Phase 1 dose escalation of ONT-10, a therapeutic MUC1 vaccine, in patients with advanced cancer. J. Immunother. Cancer.

[162] Butts C., Socinski M.A., Mitchell P.L., Thatcher N., Havel L., Krzakowski M., Nawrocki S., Ciuleanu T.E., Bosquee L., Trigo J.M. (2014). Tecemotide (L-BLP25) versus placebo after chemoradiotherapy for stage III non-small-cell lung cancer (START): A randomised, double-blind, phase 3 trial. Lancet Oncol..

[163] Gatti-Mays M.E., Redman J.M., Donahue R.N., Palena C., Madan R.A., Karzai F., Bilusic M., Sater H.A., Marte J.L., Cordes L.M. (2020). A Phase I Trial Using a Multitargeted Recombinant Adenovirus 5 (CEA/MUC1/Brachyury)-Based Immunotherapy Vaccine Regimen in Patients with Advanced Cancer. Oncologist.

[164] Quoix E., Lena H., Losonczy G., Forget F., Chouaid C., Papai Z., Gervais R., Ottensmeier C., Szczesna A., Kazarnowicz A. (2016). TG4010 immunotherapy and first-line chemotherapy for advanced non-small-cell lung cancer (TIME): Results from the phase 2b part of a randomised, double-blind, placebo-controlled, phase 2b/3 trial. Lancet Oncol..

[165] Deisseroth A., Tang Y., Zhang L., Akbulut H., Habib N. (2013). TAA/ecdCD40L adenoviral prime-protein boost vaccine for cancer and infectious diseases. Cancer Gene Ther..

[166] Tan T.J.Y., Chia J.W.K., Chong H.-S., Li X., Tan S.H., Hopkins R., Wang W.-W., Toh H.C. (2018). First-in-man study of Ad-sig-hMUC1/ecdCD40L vaccine for immunotherapy of MUC1 overexpressing epithelial cancers. J. Clin. Oncol..

